# Diet and risk of glioma: combined analysis of 3 large prospective studies in the UK and USA

**DOI:** 10.1093/neuonc/noz013

**Published:** 2019-01-23

**Authors:** Ai Seon Kuan, Jane Green, Cari M Kitahara, Amy Berrington De González, Tim Key, Gillian K. Reeves, Sarah Floud, Angela Balkwill, Kathryn Bradbury, Linda M Liao, Neal D Freedman, Valerie Beral, Siân Sweetland, The Million Women Study, the NIH-AARP study, and the PLCO study

**Affiliations:** 1 Cancer Epidemiology Unit, Nuffield Department of Population Health, University of Oxford, Oxford, UK; 2 Radiation Epidemiology Branch, Division of Cancer Epidemiology and Genetics, National Cancer Institute, Bethesda, Maryland, USA; 3 Metabolic Epidemiology Branch, Division of Cancer Epidemiology and Genetics, National Cancer Institute, Bethesda, Maryland, USA

**Keywords:** central nervous system neoplasms, diet, food and beverages, glioma, nutrition

## Abstract

**Background:**

Available evidence on diet and glioma risk comes mainly from studies with retrospective collection of dietary data. To minimize possible differential dietary recall between those with and without glioma, we present findings from 3 large prospective studies.

**Methods:**

Participants included 692 176 from the UK Million Women Study, 470 780 from the US National Institutes of Health–AARP study, and 99 148 from the US Prostate, Lung, Colorectal, and Ovarian Cancer Screening Trial. Cox regression yielded study-specific adjusted relative risks for glioma in relation to 15 food groups, 14 nutrients, and 3 dietary patterns, which were combined, weighted by inverse variances of the relative risks. Separate analyses by <5 and ≥5 years follow-up assessed potential biases related to changes of diet before glioma diagnosis.

**Results:**

The 1 262 104 participants (mean age, 60.6 y [SD 5.5] at baseline) were followed for 15.4 million person-years (mean 12.2 y/participant), during which 2313 incident gliomas occurred, at mean age 68.2 (SD 6.4). Overall, there was weak evidence for increased glioma risks associated with increasing intakes of total fruit, citrus fruit, and fiber and healthy dietary patterns, but these associations were generally null after excluding the first 5 years of follow-up. There was little evidence for heterogeneity of results by study or by sex.

**Conclusions:**

The largest prospective evidence to date suggests little, if any, association between major food groups, nutrients, or common healthy dietary patterns and glioma incidence. With the statistical power of this study and the comprehensive nature of the investigation here, it seems unlikely we have overlooked major effects of diet on risk of glioma that would be of public health concern.

**Key Points**

1. Systematic and comprehensive investigation of diet and glioma risk in 3 large prospective studies.

2. Weak or null associations between food groups, nutrients or dietary patterns and glioma risk.

3. Diet is unlikely to have major public health implications for risk of glioma.

Observational studies have reported associations of glioma risk with intakes of many different food groups and nutrients or dietary pattern scores. Findings are generally inconclusive and the vast majority of published evidence comes from studies with retrospective collection of dietary information. However, glioma may impair cognitive function and is often rapidly fatal, so evidence from retrospective glioma studies is subject to possible biases related to differential participation of cases (with glioma) and controls (otherwise healthy people) and to differential recall, especially since proxy respondents sometimes report the case’s past diet. Prospective studies have examined just 12 food groups or nutrients and there is a lack of data for many of the major components of diet.^1–17^ To provide reliable epidemiological evidence, we report here results from a systematic investigation into diet and glioma risk combining individual-participant data for more than 1.2 million adults in 3 large prospective cohort studies in the UK and the USA.

## Methods

### Study Participants, Data Collection, and Follow-Up

We used individual-participant data from the Million Women Study in the UK and the National Institutes of Health (NIH)–AARP (formerly the American Association of Retired Persons) study and the Prostate, Lung, Colorectal, and Ovarian (PLCO) Cancer Screening Trial in the USA.

In the Million Women Study, 1.3 million women aged 50–64 invited to attend the UK’s National Health Service (NHS) Breast Screening Programme were recruited in 1996–2001.^18^ A semi-quantitative dietary questionnaire was mailed to participants 3.3 years after recruitment (on average) in median year 2001 (interquartile range, 2000–2003). It was completed by 867 000 participants and provided information on intakes of about 130 foods, dietary items, and beverages. Some participants also provided dietary information through an online 24-hour dietary recall questionnaire (the Oxford WebQ) in median year 2013 (interquartile range, 2012–2015). The validity of both of the dietary questionnaires has been assessed by comparison with food diaries, and their performances were good.^19,20^ Participants are followed by electronic record linkage to the UK NHS databases for cancer registrations (coded using the tenth revision of the International Classification of Diseases [ICD-10] and the third edition of the International Classification of Diseases for Oncology [ICD-O-3]), hospital admissions, and deaths. Data in England are provided by NHS Digital and in Scotland by the Public Benefit and Privacy Panel for Health and Social Care, part of NHS Scotland.

In the NIH-AARP study, over half a million members of AARP aged 50–69 who resided in one of 6 US states (California, Florida, Pennsylvania, New Jersey, North Carolina, and Louisiana) or 2 US metropolitan areas (Atlanta, Georgia and Detroit, Michigan) were recruited in 1995–1996.^21^ Approximately 567 000 participants provided dietary information through a 124-item food frequency questionnaire (FFQ), along with other information such as lifestyle factors, at recruitment. The validity and performance of the FFQ are good.^22^ Participants were followed by probabilistic linkage to state cancer registries in the 8 original states and 3 additional states (Arizona, Nevada, and Texas), where participants most commonly moved during follow-up for cancer registrations (coded using ICD-10 and ICD-O-3). Vital status was obtained by linkage to the National Death Index and cancer registry linkage.

In the PLCO study, about 155 000 participants aged 55–74 were recruited and given a baseline questionnaire when they participated in the PLCO Screening Trial through 10 study centers across the US (Alabama, Michigan, Colorado, Hawaii, Wisconsin, Minnesota, Pennsylvania, Utah, Missouri, and Washington DC) during 1993–2001.^23,24^ About 3 years after randomization, 111 000 participants provided dietary information using an FFQ, which was virtually identical to that of the NIH-AARP study (both developed by the US National Cancer Institute [NCI]). Participants were followed via an annual study questionnaire for information on cancer diagnosis and/or death, and via National Death Index Plus searches for information on death. For every suspected cancer that was identified in the PLCO study, medical record abstraction was performed to obtain information on cancer site and morphology (coded using ICD-10 and the second edition of the ICD-O).

The design, data collection, follow-up, and data access for each study are further described in the Supplementary Material (available online) and are on the study-specific websites (www.millionwomenstudy.orghttps://dietandhealth.cancer.gov/ and https://biometry.nci.nih.gov/cdas/plco/). The Million Women Study has ethical approval from the NHS Health Research Authority (approval provided by Anglia and Oxford Multi-centre Research Ethics Committee, ref. MREC97/5/001). Women joining the study gave written consent to re-contact and to follow-up through central NHS records when completing the recruitment questionnaire. The NIH-AARP study was approved by the Special Studies Institutional Review Board of the NCI, and all study participants gave written consent by virtue of completing and returning the questionnaire. For the PLCO study, written informed consent was obtained from all study participants, and the study for human subjects research was approved by the institutional review boards at the NCI and 10 participating study centers.

### Dietary Exposure

To examine the association between diet and risk of glioma (covering major dietary components) we studied 29 food groups or nutrients (total fruit, citrus fruit, fruit juice, total vegetables, nuts, grains/cereal, red meat, processed meat, white meat (poultry), fish, eggs, dairy products, cheese, coffee, tea, carbohydrate, protein, total fat, saturated fat, monounsaturated fat, polyunsaturated fat, alcohol, fiber, carotene, vitamin C, vitamin E, folate, calcium, and cholesterol) using similar definitions across all 3 studies. We also examined the associations with risk of glioma of 3 common dietary pattern scores: the Dietary Approaches to Stop Hypertension (DASH) score, the alternate Mediterranean diet (aMED) score, and the Alternative Healthy Eating Index (AHEI).

In all 3 studies, daily intakes of the 29 food groups and nutrients were standardized to 1600 kcal/day in women and to 2000 kcal/day in men (the estimated mean daily energy intakes for women and men in the 3 prospective studies, respectively), while intakes of carbohydrate, protein, and total fat and fat subtypes were expressed as percentage energy intake from these nutrients. Participants in each study (stratified by sex) were categorized into fourths according to their energy-standardized intakes of the 29 food groups and nutrients at baseline.

The energy-standardized dietary intakes were also used to derive 3 dietary patterns: the DASH score (8 dietary components with a total of 8–40 points),^25^ the aMED score (9 dietary components with a total of 0–9 points),^26^ and the AHEI (11 dietary components with a total of 0–110 points).^27^ The scoring criteria for each dietary pattern are described further in the Supplementary Material. For DASH score and AHEI, participants in each study (stratified by sex) were categorized into fourths according to their total points. For the aMED score, participants were categorized into 4 categories: 0–2, 3–4, 5–6, and 7–9 points.

### Ascertainment of Glioma

Glioma was ascertained by combining ICD-10 codes for central nervous system (CNS) tumors (C70–C72, C75.1–C75.3, D32–D33, D35.2–35.4, D42–D43, D44.3–D44.5) and ICD-O-2 (PLCO study) or ICD-O-3 (Million Women and NIH-AARP) morphology codes for glioma (9380.3–9460.3).

### Statistical Analysis

The analysis was restricted to participants who reported dietary information. Participants were excluded in whom any invasive cancer had been diagnosed (ICD-10: C00–C97) other than non-melanoma skin cancer (ICD-10: C44) prior to dietary assessment, or if they reported an implausible energy intake through the baseline dietary questionnaire (<500 or >3500 kcal/day in women; <800 or >4200 kcal/day in men). In the Million Women Study, women were also excluded if they had a prior registration of any benign CNS tumor (ICD-10: D32–D33, D35.2–35.4, D42–D43, D44.3–D44.5) or any hospital admissions for neurofibromatosis (ICD-10: Q85.0) or tuberous sclerosis (ICD-10: Q85.1) or reported having changed their diet because of illness within 5 years prior to dietary assessment (to reduce the impact of reverse causation). The information on benign CNS tumors, neurofibromatosis, tuberous sclerosis, and changing diet was not available in the NIH-AARP and PLCO studies.

After the exclusions, study participants contributed person-years from the date of completing the dietary assessment until date of registration of glioma or other malignant cancer except non-melanoma skin cancer (also the date of any benign CNS tumor in the Million Women Study), date of death, date of loss to follow-up, or last date of follow-up (December 31, 2015 in the Million Women Study; December 31, 2011 in the NIH-AARP study; and December 31, 2009 in the PLCO study), whichever was earliest.

Taking time into the study as the underlying time variable, we used Cox regression models to estimate relative risk (RR) and 95% confidence intervals of glioma in relation to the 29 food groups or nutrients (fourths) and 3 dietary patterns (4 categories) in each of the 3 studies, and estimated linear trends for the 29 food groups or nutrients (continuous) as follows. In the NIH-AARP study and the PLCO study, linear trends were estimated using baseline dietary intake data. In the Million Women Study, linear trend was estimated using information from both baseline and remeasured dietary intakes to allow for changes in diet over time and regression dilution bias^28^; this was done by applying the remeasured intakes to the 4 dietary intake categories at baseline.

All analyses were conducted using the multivariate nutrient density method, in which energy-standardized dietary intakes (and dietary patterns derived using energy-standardized intakes) were used as the primary exposures and dietary energy intake was added into the statistical model in addition to other relevant adjustment variables.^29^ Adjustment variables in all 3 studies were height, body mass index (BMI), smoking, alcohol intake (except for the analysis of alcohol and glioma risk), level of educational attainment, and region of residence; and additionally included parity, oral contraceptive use, and use of menopausal hormones for women (categorization of all adjustment variables is described in the Supplementary Material). In the Million Women Study, analyses were stratified by year of birth and year of completing the baseline dietary assessment and were further adjusted for physical activity and social deprivation. In the NIH-AARP study, analyses were stratified by sex and were further adjusted for age, physical activity, ethnicity, and marital status. In the PLCO study, analyses were stratified by sex and were further adjusted for age, ethnicity, and marital status (information on physical activity not available in the PLCO study). In all 3 studies, participants with a missing value for any of the adjustment variables were assigned a separate category for that variable to ensure that the same participants were compared in all analyses.

Fully adjusted sex-specific log RRs of linear trend as well as log RRs comparing the fourths for all food groups and nutrients and specific categories for the 3 dietary pattern scores across the 3 studies were combined using inverse-variance weighted fixed-effect meta-analysis. Heterogeneity of results between studies was assessed by a χ^2^ test. All analyses were also stratified by follow-up time (<5 and ≥5 y) to assess potential biases related to changes of diet before ascertainment of glioma. All analyses were performed in Stata statistical software, release 15.

## Results

This analysis included a total of 1 262 104 participants, mean age 60.6 (SD 5.5) at baseline: 692 176 (women) in the UK Million Women Study, 470 780 (277 906 men, 192 874 women) in the US NIH-AARP study, and 99 148 (47 196 men, 51 952 women) in the US PLCO study (Supplementary Figure 1). Table 1 shows baseline characteristics of participants and results of follow-up for glioma for the 3 studies. When compared with participants in the Million Women Study, participants in the 2 US prospective studies were more likely to have had tertiary education and had somewhat higher BMIs, and the women were more likely to be current users of hormone replacement therapy. Participants in the 2 US studies had similar sex-specific estimated mean dietary energy intakes and percentage energy intakes from carbohydrate, protein, and total fat, whereas women in the Million Women Study reported a higher mean dietary energy intake with higher percentage energy intake from total fat and lower percentage energy intake from carbohydrate (Supplementary Table 1 shows all dietary factors assessed in this report in every study).

**Table 1:** Baseline characteristics of participants in the Million Women Study, the NIH-AARP study, and the PLCO study and results of follow-up for glioma

	Million Women Study	NIH-AARP Study		PLCO Study	
	Women	Men	Women	Men	Women
**Number of participants**	692 176	277 906	192 874	47 196	51 952
**Characteristics at baseline**					
Age, y, mean (SD)	59.4 (4.9)	61.6 (5.4)	61.3 (5.4)	65.8 (5.7)	65.3 (5.7)
Height, cm, mean (SD)	162.4 (6.5)	178.3 (7.5)	163.3 (6.9)	177.9 (6.9)	163.2 (6.5)
BMI, kg/m^2^, mean (SD)	25.9 (4.4)	27.2 (4.3)	26.8 (6.0)	27.5 (4.1)	27.0 (5.4)
Alcohol intake, drink/day, mean (SD)	0.7 (0.8)	1.1 (2.1)	0.4 (1.0)	0.9 (1.8)	0.4 (0.8)
Education—tertiary, *n* (%)	109 712 (16.2)	125 373 (46.3)	58 004 (31.1)	20 023 (42.5)	15 981 (30.8)
Race—white, non-Hispanic, *n* (%)	676 318 (99.1)	258 086 (93.9)	172 980 (91.0)	42 935 (91.0)	47 417 (91.3)
Married/living with a partner, *n* (%)	550 460 (81.0)	237 155 (85.8)	85 813 (44.9)	40 251 (85.5)	37 342 (72.0)
Strenuous exercise ≥1/week, *n* (%)	289 676 (43.0)	198 786 (71.5)	120 105 (60.3)	n/a	n/a
Current smoker, *n* (%)	84 540 (12.4)	28 028 (10.5)	26 954 (14.5)	4691 (9.9)	4377 (8.4)
Nulliparous, *n* (%)	78 093 (11.3)	n/a	28 952 (15.2)	n/a	3800 (7.3)
HRT current user, *n* (%)	189 962 (28.1)	n/a	85 766 (44.6)	n/a	27 247 (52.7)
Ever use OC, *n* (%)	420 220 (61.2)	n/a	76 152 (40.1)	n/a	28 835 (55.6)
**Daily dietary intakes, mean (SD)**					
Total energy (kcal)	1612 (430)	1974 (695)	1540 (576)	1970 (694)	1493 (541)
Carbohydrate (% energy)	47.3 (7.0)	48.1 (9.1)	51.1 (8.9)	47.1 (8.7)	50.2 (8.6)
Protein (% energy)	16.3 (2.6)	15.4 (3.1)	15.5 (3.2)	15.3 (2.9)	15.5 (2.9)
Total fat (% energy)	34.3 (6.1)	30.4 (7.6)	30.0 (7.7)	32.3 (7.4)	31.3 (7.6)
**Follow-up for glioma**					
Mean person-years of follow-up per participant	12.7	11.9	12.8	7.7	8.2
Incident glioma, *n*	1173	690	315	82	53

Abbreviations: HRT hormone replacement therapy; OC oral contraceptives.

The participants were followed for a total of 15.4 million person-years (mean 12.2 y per participant), during which 2313 incident gliomas occurred, at mean age 68.2 (SD 6.4). Numbers of gliomas registered were 1173 in the Million Women Study, 1005 (690 in men, 315 in women) in the NIH-AARP study, and 135 (82 in men, 53 in women) in the PLCO study.

**Fig. 1:**
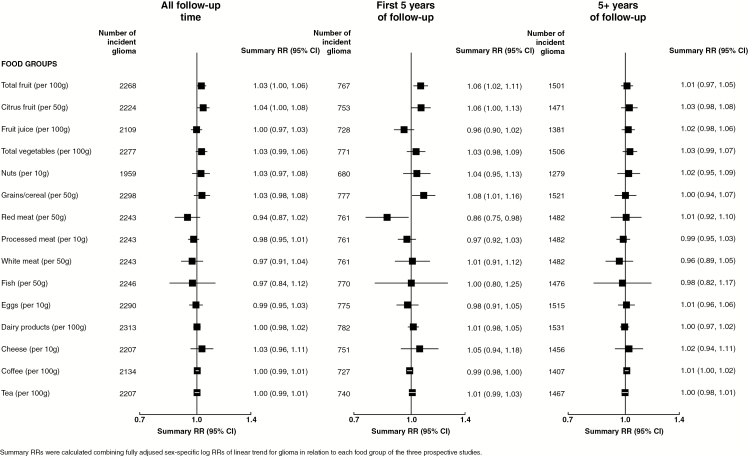
Risk of glioma in relation to increasing intakes of food groups in the Million Women Study, the NIH-AARP study, and the PLCO study.

**Fig. 2:**
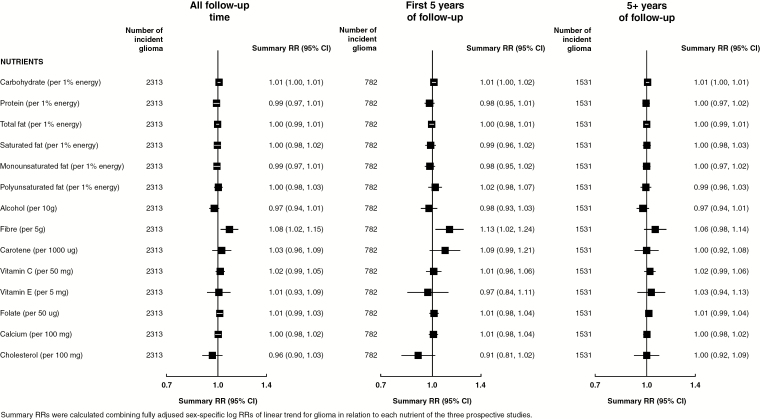
Risk of glioma in relation to increasing intakes of nutrients in the Million Women Study, the NIH-AARP study, and the PLCO study.

**Fig. 3:**
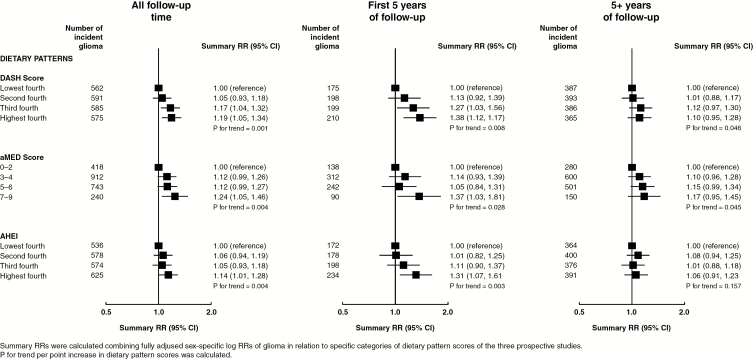
Risk of glioma in relation to dietary patterns in the Million Women Study, the NIH-AARP study, and the PLCO study.

Fig. 1 and 2 show linear trends for glioma risk associated with 29 food groups and nutrients, respectively, and Fig. 3 shows RRs associated with dietary pattern scores (categorical), each combining results from the 3 prospective studies. Overall, there was some weak evidence for increased glioma risks associated with increasing intakes of total fruit, citrus fruit, or fiber, or high versus low DASH score, aMED score, or AHEI (Figs. 1–3 left panel).

Because short-term associations of glioma risk with dietary intakes could be due to reverse causation, whereby preclinical disease affects dietary intake, results excluding the first 5 years of follow-up minimize this possibility. In analyses excluding the first 5 years of follow-up, there was little or no association between any food groups, nutrients, or dietary patterns and risk of glioma (Figs. 1–3 right panel). There was little evidence for heterogeneity of sex-specific results across the 3 studies (Supplementary Table 2). Supplementary Figure 2A–E shows sex-specific linear trends for glioma risks associated with intakes of food groups and nutrients for each of the prospective studies. Similar results were seen when the risk of glioma was estimated using intakes of food groups or nutrients in fourths rather than examining linear trends (Supplementary Table 2) and when only 3 years of follow-up were excluded (Supplementary Table 3).

## Discussion

This standardized systematic investigation into diet in 3 large prospective studies in the UK and the US shows little, if any, evidence for an association between diet and risk of glioma. This is particularly evident in analyses that excluded the first 5 years of follow-up, which aimed to reduce any biases related to changes of diet associated with prediagnostic manifestation of glioma. There was no strong evidence for heterogeneity of results across the studies.

Our analysis contributes substantially to the current literature of diet and risk of glioma, by providing prospective evidence as well as including the largest number of glioma cases published to date. The current report includes more than twice as many glioma cases as all other published prospective studies combined. For the 12 dietary factors with previously published prospective evidence (total fruit, citrus fruit, fruit juice, total vegetables, red meat, processed meat, alcohol, coffee, tea, carotene, vitamin C, and vitamin E), the results presented here are consistent with the null and nonsignificant results from previously published prospective studies.^1–17^ For the other 17 food groups or nutrients as well as the 3 dietary pattern scores examined here, our analysis provided the first prospective evidence. The observed small associations between intakes of total fruit, citrus fruit, fiber, or healthy dietary pattern scores and glioma risk were attenuated toward the null after excluding the first 5 years of follow-up and may well be due to reverse causation bias resulting from dietary changes associated with preclinical glioma.

Some (but not all) studies with retrospective collection of dietary data reported significant associations of glioma risk with various dietary factors (decreased glioma risks with intakes of citrus fruit,^30,31^ total vegetables,^32^ alcohol,^33^ coffee,^34^ tea,^34^ fiber,^35^ carotene,^35,36^ vitamin C,^36^ vitamin E,^32^ and DASH score^37^; and increased glioma risks with intakes of grains/cereal,^31,38^ processed meat,^39^ and eggs^31,38^). However, it is unclear to what extent these results were subject to differential reporting of diet as well as biases related to differential participation of individuals with and without glioma. Besides, because glioma is often rapidly fatal, the reliance on proxy respondents in the 8 retrospective studies that reported this information was high (8–76%),^30,31,35,40–44^ making the results from such studies of glioma difficult to interpret.

The present study has several strengths. With the prospective nature of data collection and the large number of glioma cases occurring during participants’ follow-up, we were able to perform analyses stratified by follow-up time to assess potential reverse causation and to minimize it by excluding the first 5 years of follow-up. The standardized methods in the present individual participant data meta-analysis reduce heterogeneity between studies due to differences in categorization of dietary exposures, definition of glioma outcomes, as well as statistical models, which are major limitations of meta-analyses of published evidence. The systematic investigation into diet with glioma risks avoids biases related to selective reporting commonly seen in nutritional epidemiology studies.^45^

The main limitation of the present analyses is that self-reported dietary intakes that are collected at baseline are subject to errors, including random measurement error, which may appreciably attenuate any possible association examined in epidemiological analysis due to regression dilution.^28,46^ While the analyses in the Million Women Study allowed for regression dilution using remeasured dietary intakes, analyses in the NIH-AARP study and the PLCO study did not because there was no remeasured dietary information. Also, small effects of diet may have been undetected despite this study being by far the largest single analysis for diet and risk of glioma. When comparing the extreme fourths (or 4 categories) of dietary factors, this study has 80% power with 95% significance to detect an observed RR of ≤0.85 or ≥1.18 in analyses of all follow-up time and an RR of ≤0.82 or ≥1.22 in analyses excluding the first 5 years of follow-up. While it is possible that small but genuine associations were not detected, associations of small magnitude are unlikely to be of major public health concern unless an extreme intake of specific dietary items or nutrients is particularly common. Lack of histopathology data is another limitation of the current study.

The prospective evidence based on the largest number of incident glioma cases to date suggests that there is little, if any, evidence of a strong effect of diet, including major food groups, nutrients, and common healthy dietary patterns, on risk of glioma. With the rarity of glioma, the statistical power, and the comprehensive nature of the investigation here, it seems unlikely we have overlooked major effects of diet on risk of glioma that would be of public health concern. The results do not, however, exclude the possibility of a role of diet in disease prognosis, which perhaps warrants further study.

## Funding

The work in the Million Women Study was supported by the Medical Research Council UK and Cancer Research UK. The work in the NIH-AARP study was supported (in part) by the Intramural Research Program of the NIH, National Cancer Institute. The work in the PLCO study was supported by the Intramural Research Program of the Division of Cancer Epidemiology and Genetics and by contracts from the Division of Cancer Prevention, NCI, NIH, and Department of Health and Human Services. The sponsors had no role in the design of the study; the collection, analysis, or interpretation of the data; the writing of the manuscript; or the decision to submit the manuscript for publication.

## 

**Conflict of interest statement.** None declared.

## 

**Authorship statement.** ASK, JG, CK, AB, VB, and SS conceived and designed the analysis. ASK conducted statistical analysis under the supervision of JG, CK, AB, and SS. All authors helped in interpreting the findings. ASK wrote the first draft of the manuscript. All authors contributed toward subsequent revisions and approved the submitted manuscript.

## Supplementary Material

noz013_suppl_Supplementary_Materials
