# Supplementary material for: Diet and risk of glioma: combined analysis of 3 large prospective studies in the UK and USA
Source: Neuro Oncol. 2019 Jan 23;21(7):944–52. doi: 10.1093/neuonc/noz013 (PMC6620629; doi:10.1093/neuonc/noz013)
Supplement: noz013_suppl_Supplementary_Materials [file noz013_suppl_supplementary_materials.docx]

**SUPPLEMENTARY MATERIALS**

**Diet and risk of glioma: combined analysis of three large prospective studies in the UK and USA**

Ai Seon Kuan, Jane Green, Cari M. Kitahara, Amy Berrington de González, Tim Key, Gillian Reeves, Sarah Floud, Angela Balkwill, Kathryn Bradbury, Linda M. Liao, Neal D. Freedman, Valerie Beral, and Siân Sweetland on behalf of collaborators of the Million Women Study, the NIH-AARP Study, and the PLCO Study

**SUPPLEMENTARY METHODS**

**The Million Women Study**

*Study design, data collection, and follow-up*

The Million Women Study in the UK is an open-ended ongoing study of the health of women, with prospective collection of data.^1^ More than 1.3 million women aged 50–64 who were invited to attend the National Health Service (NHS) Breast Screening Programme in England and Scotland during 1996–2001 were recruited into the study.

At recruitment, women completed the recruitment questionnaire which asked about various characteristics including socio-demographic, anthropometric, lifestyle, and reproductive factors. Since recruitment, four postal re-survey questionnaires (the 3-, 8-, 12-, and 15-year re-survey questionnaires) have been sent to surviving participants every 3–5 years to update information collected previously which may have changed over time, and to collect new information such as diet and social participation. The 3-year re-survey questionnaire was completed by approximately 867,100 women in median year 2001 (IQR 2000–2003), and provided first dietary information on intakes of about 120 foods, dietary items, and beverages, which is the baseline information for diet used here. The repeatability of most of the dietary intake questions and the performance of the questionnaire for estimating nutrient intakes when compared against a 7-day diet diary were good.^2^ Since 2010, surviving women who provided valid email addresses were invited to complete, the Oxford WebQ, an online 24-hour recall dietary questionnaire which provide repeat measures of dietary information used here. Good agreements were found for the great majority of dietary intakes captured by the Oxford WebQ when compared to an interviewer-administered 24-hour dietary recall assessment.^3^ Intakes of total energy and nutrients were calculated by multiplying the frequency of intakes of particular dietary item by the specified portion size with the energy and nutrient composition of that particular dietary item.^2,3^

Women are followed up through electronic record linkage to the UK NHS databases for cancer registrations (cancer site coded using the 10th revision of the International Classification of Diseases [ICD-10] and tumour morphology coded using the 3rd edition of the International Classification of Diseases for Oncology [ICD-O-3]), hospital admissions (coded using ICD-10), and deaths (coded using ICD-10). Data in England are provided by the NHS Digital and in Scotland by the Public Benefit and Privacy Panel for Health and Social Care, part of the NHS Scotland. Follow-up remains virtually complete after 20 years, with about 1% loss to follow-up (up to 31 December 2015).^1^ The sensitivities of the cancer registries for cancer case ascertainment were shown to be extremely good in both England and Scotland.^4,5^ The endpoints included in the current analysis are first registration of glioma (ICD-10 codes C70–C72, C75.1–C75.3, D32–D33, D35.2–35.4, D42–D43, D44.3–D44.5 and ICD-O-3 codes 9380.3–9460.3) in the cancer registers. More information on study is available at the Million Women Study website (www.millionwomenstudy.org).

*Statistical analysis*

Women were excluded under standard rules if they had no corresponding follow-up information (n=18), or had missing information on date of birth (n=544), date of follow-up (n=9), or date of dietary assessment (n=6). Women were also excluded if they had, prior to the baseline dietary assessment, any cancer or any benign CNS tumours (n=45,953) (ICD-10 C00–C97 [except non-melanoma skin cancer ICD-10 C44] and ICD-10 D32–D33, D35.2–35.4, D42–D43, D44.3–D44.5, respectively], or any hospital admission diagnosis of neurofibromatosis or tuberous sclerosis (n=181) (ICD-10 Q85.0 and ICD-10 Q85.1, respectively); or reported having changed their diet because of illness within 5 years prior to the baseline dietary assessment (n=123,937); or reported an implausible energy intake (<500 or >3500 kcal/day) in the baseline dietary assessment (n=4,276). The remaining women (N=692,176) contributed person-years from the date of baseline dietary assessment until the earliest of the date of registration of glioma or any CNS tumour or any cancer (except non-melanoma skin cancer), date of death, date of loss to follow-up, or the last date of follow-up (31 December 2015).

Relative risk (RRs) and 95% confidence intervals (CIs) of glioma in relation to the 29 food groups or nutrients and 3 dietary patterns were estimated using Cox regression models with time in the study as the underlying time variable, stratified by year of birth (≤1930, 1931, 1932, …, 1948, 1949, ≥1950) and year of completing the baseline dietary assessment (≤2000, 2001, 2002, 2003, ≥2004). Analyses were adjusted for dietary energy intake (fourths), height (<160, 160–164.9, 165–169.9, ≥170 cm), body mass index (<25, 25–29.9, ≥30 kg/m^2^), strenuous exercise (never/rarely, <1, ≥1 times per week), social deprivation (thirds based on Townsend deprivation index), level of educational attainment (<12 years, secondary, tertiary – had a university degree), smoking status (never, past, current), alcohol intake (<1, ≥1 drinks per day) (except of the analysis of alcohol and glioma risk), parity (nulliparous, parous), oral contraceptive use (never, ever), use of menopausal hormones (never, past, current), and region of residence (nine geographical regions in England, plus Scotland).

**The NIH-AARP Study**

*Study design, data collection, and follow-up*

The National Institutes of Health (NIH)-AARP (formerly the American Association of Retired Persons) Study in the US was set up to investigate the association between diet and cancer.^6^ In 1995–1996, Over half a million members of the AARP aged 50–69 who resided in one of the 6 states (California, Florida, Pennsylvania, New Jersey, North Carolina, Louisiana) and 2 metropolitan areas (Atlanta, Georgia and Detroit, Michigan) in the US were recruited into the study.

At recruitment, approximately 567,100 participants provided dietary information through a 124-item food frequency questionnaire (FFQ), along with other information including on socio-demographic, anthropometric, lifestyle, and reproductive factors. Energy and nutrient intakes were calculated according to the intake information of a particular dietary item and energy and nutrient composition of that particular item.^7,8^ The performance of the 124-item FFQ for estimating nutrient intakes when compared against two 24-hour dietary recalls was good.^7^ Since recruitment, two follow-up questionnaires were sent to surviving participants to collect information on various characteristics including early life diet and retirement but none of the information collected using these two follow-up questionnaires was used here.

Participants were followed up through electronic record linkage to National Change of Address database for information on changes in addresses and the US Social Security Administration’s Death Master File for death registration.^9^ In the first 3 years of follow-up, only about 2% of participants were lost to follow-up due to emigration.^9^ Surviving participants who did not move outside the study areas during the follow-up period were followed up through electronic record linkage to state cancer registries for cancer registrations (coded using ICD-10 and ICD-O-3). The sensitivities of the cancer registries for cancer case ascertainment were shown to be very good.^9^ The endpoints included in the current analysis are first registrations of glioma (ICD-10 codes C70–C72 and C75.1–C75.3 and ICD-O-3 codes 9380.3–9460.3) in the cancer registries. More information on study is available at the NIH-AARP Study website (https://dietandhealth.cancer.gov/).

*Statistical analysis*

Participants were excluded under standard rules if information about participants was provided by proxy respondents (n=15,760), or they had missing information on follow-up (n=56), or had conflicted information on sex (n=162), or had conflicted information on cancer diagnosis (n=4,255) (e.g. had a cancer diagnosis from death records but not cancer registries). Participants were also excluded if they had, prior to the baseline dietary assessment, any cancer (n=51,346) (ICD-10 C00–C97 [except non-melanoma skin cancer ICD-10 C44]) or reported an implausible energy intake (<500 or >3500 kcal/day for women; <800 or >4200 kcal/day for men) in the baseline dietary assessment (n=24,039). The remaining participants (N=470,780) contributed person-years from the date of baseline dietary assessment until the earliest of the date of registration of glioma or any cancer (except non-melanoma skin cancer), date of death, date of loss to follow-up ^9^, or the last date of follow-up (31 December 2011).

Sex-specific RRs and 95% CIs of glioma in relation to the 29 food groups or nutrients and 3 dietary patterns were estimated using Cox regression models with time in the study as the underlying time variable. All analyses were adjusted for age (continuous), sex-specific dietary energy intake (fourths), sex-specific height (<160, 160–164.9, 165–169.9, ≥170 cm for women; <175, 175–179.9, 180–184.9, ≥185 cm for men), body mass index (<25, 25–29.9, ≥30 kg/m^2^), strenuous exercise (never/rarely, <1, ≥1 times per week), ethnicity (non-Hispanic Whites, others), marital status(married/living with a partner, others), level of educational attainment (<12 years, secondary, tertiary – had a university degree), smoking status (never, past, current), alcohol intake (<1, ≥1 drinks per day) (except of the analysis of alcohol and glioma risk), and region of residence (California, Florida, Pennsylvania, New Jersey, North Carolina, Louisiana, Atlanta, and Detroit); with additional adjustment for parity (nulliparous, parous), oral contraceptive use (never, ever), and use of menopausal hormones (never, past, current) in women.

**The PLCO Study**

*Study design, data collection, and follow-up*

The Prostate, Lung, Colorectal, and Ovarian Cancer Screening Trial [PLCO Study]) in the US was designed to assess the effect of cancer screening tests on mortality for cancers of the prostate, lung, colon and rectum, and ovary.^10^ The study has also investigated risks of cancers including glioma in relation to many other factors such as diet. In 1993–2001, about 155,000 participants were recruited through 10 study centres across the US (Alabama, Michigan, Colorado, Hawaii, Wisconsin, Minnesota, Pennsylvania, Utah, Missouri, and Washington D.C.) and randomised.^11^

At recruitment, participants completed the baseline questionnaire which asked about various characteristics including socio-demographic, anthropometric, lifestyle, and reproductive factors. About 3 years after randomisation, approximately 111,000 participants provided dietary information using a 124-item FFQ, the Diet History Questionnaire.^11^ Energy and nutrient intakes were calculated according to the intake information of a particular dietary item and energy and nutrient composition of that particular item.^7,8^ Because the Diet History Questionnaire is virtually identical to the FFQ used by the NIH-AARP Study (both developed by the US National Cancer Institute), it can be assumed that the performance of the Diet History Questionnaire will be similar to the FFQ in the NIH-AARP Study. Since recruitment, three other questionnaires were also sent to subsets participants to collect information such as on diet (intervention arm participants only) and cancer screening exams but none of the information collected using these three questionnaires was used here.^11^

Participants were followed via an annual study questionnaire, the Annual Study Updates, for information on cancer diagnosis and/or death. About 96% of the expected 1.7 million Annual Study Updates were completed and returned.^11^ Participants were also followed via the National Death Index Plus searches for information on death. For every suspected cancer that was identified in the PLCO Study, medical record abstraction was performed to obtain information on cancer site and morphology (coded using ICD-10 and the 2nd edition of the international classification of diseases for oncology [ICD-O-2]). The endpoints included in the current analysis are first ascertained glioma (ICD-10 codes C70–C72 and C75.1–C75.3 and ICD-O-2 codes 9380.3–9460.3). More information on study is available at the PLCO Study website (https://biometry.nci.nih.gov/cdas/plco/)

*Statistical analysis*

In the PLCO Study, participants were excluded under standard rules if their had missing information on follow-up time (n=55). Participants were also excluded if they had, prior to the baseline dietary assessment, any cancer (n=9,636) (ICD-10 C00–C97 [except non-melanoma skin cancer ICD-10 C44]) or reported an implausible energy intake (<500 or >3500 kcal/day for women; <800 or >4200 kcal/day for men) in the baseline dietary assessment (n=2,573). The remaining participants (N=99,148) contributed person-years from the date of baseline dietary assessment until the earliest of the date of ascertainment of glioma or any cancer (except non-melanoma skin cancer), date of death, date of loss to follow-up (about 4% by the end of follow-up)^11^, or the last date of follow-up (31 December 2009).

Sex-specific RRs and 95% CIs of glioma in relation to the 29 food groups or nutrients and 3 dietary patterns were estimated using Cox regression models with time in the study as the underlying time variable. All analyses were adjusted for age (continuous), sex-specific dietary energy intake (fourths), sex-specific height (<160, 160–164.9, 165–169.9, ≥170 cm for women; <175, 175–179.9, 180–184.9, ≥185 cm for men), body mass index (<25, 25–29.9, ≥30 kg/m^2^), ethnicity (non-Hispanic Whites, others), marital status(married/living with a partner, others), level of educational attainment (<12 years, secondary, tertiary – had a university degree), smoking status (never, past, current), alcohol intake (<1, ≥1 drinks per day) (except of the analysis of alcohol and glioma risk), and region of residence (Alabama, Michigan, Colorado, Hawaii, Wisconsin, Minnesota, Pennsylvania, Utah, Missouri, and Washington D.C.); with additional adjustment for parity (nulliparous, parous), oral contraceptive use (never, ever), and use of menopausal hormones (never, past, current) in women.

The covariates included in the final statistical model were chosen not only based on results from statistical tests that assessed model fit but also a review of literature regarding sociodemographic, anthropometric, and lifestyle factors that were shown to be associated with glioma and cancer incidence in general, as well as standard adjustment factors for each cohort. Results from the three cohorts were combined using individual participant data meta-analysis, so results were adjusted for similar sets of sociodemographic, anthropometric, and lifestyle factors across the three cohorts. This approach reduced between-study heterogeneity and minimized the influence of any potential confounding factors on the association between diet and glioma risks, although it may result in overfitting the models particularly in the PLCO cohort with fewer participants and glioma cases.

**Scoring criteria for the dietary pattern scores**

*The Dietary Approaches to Stop Hypertension*

The Dietary Approaches to Stop Hypertension (DASH) score measures adherence to the recommended dietary intakes to control and prevent hypertension promoted by the US National Heart, Lung, and Blood Institute of the National Institutes of Health.^12^ The DASH score measures adherence to the consumption of 8 dietary components, which have a range from 1 point to 5 points each.^12^ The total score for the DASH score ranges from 8 point (non-adherence) to 40 points (perfect adherence). For each of the following five components (total fruit and fruit juice, non-legume vegetables, nuts and legumes, dairy products, whole grains), participants were categorised into fifths according to their dietary intakes and scores of 1 to 5 points were assigned based on their ranking in fifths (e.g. 1 point for the lowest fifth, 5 points for the highest fifth). Scoring was reversed for the remaining three components: sugar-sweetened beverages, red and processed meats, and sodium. In the Million Women Study, scoring for sodium intake was based on added sodium, as asked in the question ‘how often do you add salt to food’ (5 points for never, 3 points for sometimes, 2 points for usually, and 1 point for always add salt to food).

*The alternate Mediterranean diet score*

The Mediterranean diet score measures adherence to the Mediterranean diet.^13^ The alternate Mediterranean diet (aMED) score is a modified version of the original score, which includes only dietary components that have been consistently shown to be associated with risks of chronic diseases.^14,15^ The aMED score measures adherence to the consumption of nine dietary components with a possible range from 0 to 9 points.^14^ For each of seven components (fish, legumes, whole grains, non-legume vegetables, total fruit and fruit juice, nuts, ratio of monounsaturated fat to saturated fat) 1 point was assigned for intakes equal to or greater than the sex-specific median, and 0 points otherwise. Scoring was reversed for red and processed meats, so that 1 point was assigned for intakes below the sex-specific median, and 0 points otherwise. For alcohol intake, 1 point was assigned for moderate intakes (5–15g/day in women; 10–25g/day in men), and 0 points otherwise. Participants were categorised into 4 groups (0–2, 3–4, 5–6, 7–9 points) according to their total scores.

*The alternative Healthy Eating Index*

The 2010 Healthy Eating Index (HEI) measures the adherence to the recommended dietary intakes based on the 2010 Dietary Guidelines for Americans developed by the US Department of Agriculture.^16^ The alternative Healthy Eating Index (AHEI) is a modified version of the HEI-2010 which includes only dietary components that have been consistently shown to be associated with risks of chronic diseases.^17^ The AHEI comprises eleven components, which have a range from 0 to 10 points each. The total score for the AHEI ranges from 0 points (non-adherence) to 110 points (perfect adherence). For each of the following six components, intakes were scored proportionally from 0 to 10 points according to the range of recommended intakes: non-legume vegetables (0–5 servings/day), total fruit (0–4 servings/day), whole grains (0–75g/day in women; 0–90g/day in men), nuts and legumes (0–1 serving/day), energy from intake of polyunsaturated fat excluding omega 3 fatty acids (2%–10%/day), and omega-3 fatty acids (0–250mg/day); intakes below the recommended range were assigned with 0 points while intakes above the range were assigned with 10 points. Scoring was reversed for each of the following three components: sugar-sweetened beverages (0–1 serving/day), red and processed meats (0–1.5 servings/day), and energy from intake of trans-fat (0.5%–4%/day). For alcohol, 10 points were assigned to moderate intake (0.5–1.5 drinks/day in women; 0.5–2.0 drinks/day in men); intakes were scored proportionally from 10 to 0 for intakes between 1.5 and 2.5 drinks/day in women and 2.0 and 3.5 drinks/day in men; intakes <0.5 drinks/day in both men and women was assigned 2.5 points; and intakes greater than 2.5 drinks/day in women and 3.5 drinks/day in men were assigned 0 point; For sodium, participants were usually categorised into tenths according to sodium intake and score was reversely assigned based on the rank of the tenths. In the Million Women Study, scoring for sodium was based on added salt (10 points for never, 6.7 points for sometimes, 3.3 points for usually, and 0 point for always add salt to food)

**Supplementary Table 1** Mean dietary intakes for all participants and participants with glioma in the Million Women Study, the NIH-AARP Study, and the PLCO Study

| **Daily dietary intakes (mean)** | **Million Women Study** |  | **NIH-AARP**  **Study** | |  | **PLCO**  **Study** | |
| --- | --- | --- | --- | --- | --- | --- | --- |
|  | **Women** |  | **Men** | **Women** |  | **Men** | **Women** |
| **Number of women** | 692176 |  | 277906 | 192874 |  | 47196 | 51952 |
| **Total fruit, g** | 206.8 (150.4) |  | 244.5 (214.3) | 257.1 (201.0) |  | 177.0 (146.1) | 210.2 (151.9) |
| **Citrus fruit, g** | 44.4 (49.5) |  | 46.8 (74.0) | 51.1 (74.8) |  | 28.6 (43.5) | 33.2 (45.1) |
| **Fruit juice, g** | 106.5 (94.3) |  | 147.7 (195.3) | 117.7 (163.7) |  | 108.3 (148.2) | 101.4 (132.3) |
| **Total vegetables, g** | 122.0 (84.2) |  | 255.2 (162.9) | 254.3 (163.0) |  | 238.0 (159.2) | 254.7 (158.3) |
| **Nuts, g** | 5.4 (9.4) |  | 6.6 (10.6) | 3.9 (7.0) |  | 11.4 (15.3) | 8.1 (12.1) |
| **Grains/cereal, g** | 67.6 (44.6) |  | 44.0 (59.2) | 39.2 (52.4) |  | 49.5 (63.7) | 46.4 (56.2) |
| **Red meat, g** | 30.3 (25.0) |  | 76.0 (44.4) | 47.1 (30.9) |  | 77.6 (43.2) | 47.3 (28.8) |
| **Processed meat, g** | 11.4 (12.4) |  | 24.0 (21.4) | 13.2 (14.0) |  | 22.5 (18.9) | 12.4 (12.2) |
| **White meat, g** | 21.9 (17.7) |  | 64.2 (47.4) | 57.5 (43.4) |  | 52.8 (42.7) | 53.4 (42.6) |
| **Fish, g** | 34.0 (22.9) |  | 21.9 (21.6) | 18.0 (19.0) |  | 17.0 (18.5) | 15.6 (16.6) |
| **Eggs, g** | 17.9 (14.3) |  | 12.5 (15.4) | 9.5 (12.4) |  | 15.6 (18.8) | 11.0 (14.6) |
| **Dairy products, g** | 334.6 (151.0) |  | 303.4 (298.1) | 278.5 (265.8) |  | 288.5 (283.7) | 255.5 (247.5) |
| **Cheese, g** | 19.3 (13.8) |  | 4.3 (7.3) | 3.6 (6.5) |  | 9.3 (12.8) | 7.6 (11.0) |
| **Coffee, g** | 472.4 (424.5) |  | 909.2 (792.5) | 812.2 (797.3) |  | 1101.2 (1052.2) | 844.5 (893.6) |
| **Tea, g** | 745.6 (546.5) |  | 265.7 (410.8) | 346.5 (502.1) |  | 275.6 (552.5) | 315.6 (548.5) |
| **Carbohydrate, %E** | 47.3 (7.0) |  | 48.1 (9.1) | 51.1 (8.9) |  | 47.1 (8.7) | 50.2 (8.6) |
| **Protein, %E** | 16.3 (2.6) |  | 15.4 (3.1) | 15.5 (3.2) |  | 15.3 (2.9) | 15.5 (2.9) |
| **Total fat, %E** | 34.3 (6.1) |  | 30.4 (7.6) | 30.0 (7.7) |  | 32.3 (7.4) | 31.3 (7.6) |
| **Saturated fat, %E** | 12.0 (3.7) |  | 9.5 (2.9) | 9.3 (2.9) |  | 10.4 (3.0) | 9.8 (2.9) |
| **Monounsaturated fat, %E** | 12.5 (2.8) |  | 11.6 (3.1) | 11.1 (3.1) |  | 12.3 (3.1) | 11.6 (3.1) |
| **Polyunsaturated fat, %E** | 6.4 (1.9) |  | 6.9 (2.1) | 7.2 (2.4) |  | 7.1 (2.0) | 7.4 (2.3) |
| **Alcohol, g** | 5.4 (7.7) |  | 14.2 (23.3) | 5.9 (12.7) |  | 12.6 (20.4) | 5.5 (11.0) |
| **Fibre, g** | 13.5 (4.1) |  | 13.7 (5.2) | 12.0 (4.4) |  | 13.1 (4.6) | 12.1 (4.0) |
| **Carotene, ug** | 1317.7 (732.2) |  | 897.9 (808.2) | 1031.2 (931.2) |  | 748.1 (622.5) | 891.0 (698.3) |
| **Vitamin C, mg** | 94.2 (47.1) |  | 169.5 (103.0) | 162.8 (92.6) |  | 145.6 (83.0) | 150.9 (76.9) |
| **Vitamin E, mg** | 9.0 (3.3) |  | 9.7 (4.0) | 8.3 (3.1) |  | 8.5 (3.1) | 7.8 (2.9) |
| **Folate, ug** | 247.4 (59.8) |  | 565.2 (178.5) | 472.3 (140.7) |  | 534.8 (176.5) | 457.9 (138.3) |
| **Calcium, mg** | 823.5 (210.1) |  | 826.2 (345.0) | 735.8 (304.0) |  | 840.7 (329.6) | 734.6 (281.3) |
| **Cholesterol, mg** | 215.4 (72.7) |  | 224.0 (93.7) | 170.3 (73.3) |  | 247.7 (109.0) | 182.5 (83.6) |
| **DASH score** | 22.8 (5.2) |  | 24.3 (4.7) | 24.5 (4.7) |  | 24.0 (4.7) | 24.1 (4.7) |
| **aMED score** | 4.0 (1.8) |  | 4.2 (1.8) | 4.1 (1.7) |  | 4.2 (1.8) | 4.1 (1.7) |
| **AHEI** | 47.6 (12.1) |  | 52.6 (10.2) | 53.1 (9.6) |  | 57.2 (9.0) | 58.4 (8.6) |
| **Total energy intake, kcal** | 1611.7 (430.0) |  | 1974.0 (694.7) | 1540.6 (575.6) |  | 1970.2 (694.4) | 1492.9 (541.0) |

%E percentage energy intake

**Supplementary Table 2** Risk of glioma in relation to intakes of food groups and nutrients and dietary patterns in the Million Women Study, the NIH-AARP Study, and the PLCO Study

|  |  | **All follow-up time** | | | | | |  | **First 5 years of follow-up** | | | | | |  | **5+ years of follow-up** | | | | | |
| --- | --- | --- | --- | --- | --- | --- | --- | --- | --- | --- | --- | --- | --- | --- | --- | --- | --- | --- | --- | --- | --- |
| **Dietary factor** | **Intake category** | **No. of cases** | **Summary RR (95% CI)** | **P value** | **Heterogeneity** | | |  | **No. of cases** | **Summary RR (95% CI)** | **P value** | **Heterogeneity** | | |  | **No. of cases** | **Summary RR (95% CI)** | **P value** | **Heterogeneity** | | |
|  |  |  |  |  | **I^2^ (%)** | **Χ^2^** | **P value** |  |  |  |  | **I^2^ (%)** | **Χ^2^** | **P value** |  |  |  |  | **I^2^ (%)** | **Χ^2^** | **P value** |
| **Total fruit** | Lowest fourth | 534 | 1.00 (reference) | n/a | n/a | n/a | n/a |  | 168 | 1.00 (reference) | n/a | n/a | n/a | n/a |  | 366 | 1.00 (reference) | n/a | n/a | n/a | n/a |
|  | Second fourth | 527 | 0.96 (0.85–1.08) | 0.499 | 0.0 | 3.0 | 0.556 |  | 173 | 1.00 (0.80–1.24) | 0.978 | 29.8 | 5.7 | 0.223 |  | 354 | 0.94 (0.81–1.09) | 0.423 | 0.0 | 3.6 | 0.468 |
|  | Third fourth | 572 | 1.03 (0.91–1.17) | 0.605 | 0.0 | 2.9 | 0.579 |  | 209 | 1.17 (0.95–1.45) | 0.132 | 0.0 | 1.7 | 0.793 |  | 363 | 0.97 (0.83–1.12) | 0.667 | 0.0 | 2.6 | 0.626 |
|  | Highest fourth | 635 | 1.17 (1.03–1.32) | 0.012 | 10.6 | 4.5 | 0.346 |  | 217 | 1.23 (0.99–1.52) | 0.056 | 14.5 | 4.7 | 0.322 |  | 418 | 1.14 (0.98–1.32) | 0.090 | 0.0 | 1.0 | 0.917 |
|  | *Linear trend (per 100g)* | *2268* | *1.03 (1.00–1.06)* | *0.048* | *28.5* | *5.6* | *0.232* |  | *767* | *1.06 (1.02–1.11)* | *0.008* | *30.2* | *5.7* | *0.221* |  | *1501* | *1.01 (0.97–1.05)* | *0.562* | *0.0* | *3.5* | *0.481* |
|  |  |  |  |  |  |  |  |  |  |  |  |  |  |  |  |  |  |  |  |  |  |
| **Citrus fruit** | Lowest fourth | 605 | 1.00 (reference) | n/a | n/a | n/a | n/a |  | 197 | 1.00 (reference) | n/a | n/a | n/a | n/a |  | 408 | 1.00 (reference) | n/a | n/a | n/a | n/a |
|  | Second fourth | 418 | 0.87 (0.77–0.99) | 0.039 | 0.0 | 2.5 | 0.638 |  | 142 | 0.89 (0.72–1.11) | 0.310 | 0.0 | 0.9 | 0.927 |  | 276 | 0.86 (0.74–1.01) | 0.072 | 14.2 | 4.7 | 0.324 |
|  | Third fourth | 584 | 1.06 (0.94–1.19) | 0.337 | 0.0 | 1.5 | 0.826 |  | 196 | 1.07 (0.87–1.31) | 0.520 | 0.0 | 1.2 | 0.882 |  | 388 | 1.05 (0.91–1.21) | 0.473 | 0.0 | 3.7 | 0.443 |
|  | Highest fourth | 617 | 1.13 (1.00–1.26) | 0.043 | 0.0 | 1.1 | 0.898 |  | 218 | 1.16 (0.95–1.42) | 0.144 | 21.3 | 5.1 | 0.279 |  | 399 | 1.11 (0.96–1.28) | 0.148 | 0.0 | 3.4 | 0.492 |
|  | *Linear trend (per 50g)* | *2224* | *1.04 (1.00–1.08)* | *0.043* | *4.9* | *4.2* | *0.379* |  | *753* | *1.06 (1.00–1.13)* | *0.035* | *0.0* | *1.0* | *0.911* |  | *1471* | *1.03 (0.98–1.08)* | *0.309* | *35.9* | *6.2* | *0.182* |
|  |  |  |  |  |  |  |  |  |  |  |  |  |  |  |  |  |  |  |  |  |  |
| **Fruit juice** | Lowest fourth | 514 | 1.00 (reference) | n/a | n/a | n/a | n/a |  | 187 | 1.00 (reference) | n/a | n/a | n/a | n/a |  | 327 | 1.00 (reference) | n/a | n/a | n/a | n/a |
|  | Second fourth | 550 | 1.05 (0.93–1.19) | 0.421 | 0.0 | 0.7 | 0.947 |  | 185 | 0.97 (0.79–1.19) | 0.759 | 0.0 | 0.6 | 0.959 |  | 365 | 1.10 (0.95–1.28) | 0.220 | 0.0 | 0.6 | 0.961 |
|  | Third fourth | 519 | 0.97 (0.86–1.10) | 0.646 | 1.4 | 4.1 | 0.398 |  | 182 | 0.92 (0.74–1.13) | 0.408 | 27.0 | 5.5 | 0.242 |  | 337 | 1.00 (0.86–1.17) | 0.956 | 0.0 | 3.5 | 0.481 |
|  | Highest fourth | 526 | 0.98 (0.87–1.11) | 0.775 | 0.0 | 2.3 | 0.680 |  | 174 | 0.90 (0.73–1.12) | 0.347 | 11.8 | 4.5 | 0.338 |  | 352 | 1.03 (0.88–1.20) | 0.740 | 0.0 | 1.2 | 0.879 |
|  | *Linear trend (per 100g)* | *2109* | *1.00 (0.97–1.03)* | *0.879* | *0.0* | *2.3* | *0.685* |  | *728* | *0.96 (0.90–1.02)* | *0.160* | *0.0* | *2.1* | *0.718* |  | *1381* | *1.02 (0.98–1.06)* | *0.355* | *0.0* | *1.5* | *0.821* |
|  |  |  |  |  |  |  |  |  |  |  |  |  |  |  |  |  |  |  |  |  |  |
| **Total vegetables** | Lowest fourth | 504 | 1.00 (reference) | n/a | n/a | n/a | n/a |  | 167 | 1.00 (reference) | n/a | n/a | n/a | n/a |  | 337 | 1.00 (reference) | n/a | n/a | n/a | n/a |
|  | Second fourth | 580 | 1.12 (0.99–1.26) | 0.066 | 47.3 | 7.6 | 0.108 |  | 194 | 1.13 (0.91–1.39) | 0.265 | 39.9 | 6.7 | 0.155 |  | 386 | 1.12 (0.96–1.29) | 0.146 | 0.0 | 3.8 | 0.438 |
|  | Third fourth | 598 | 1.15 (1.02–1.29) | 0.024 | 0.0 | 1.7 | 0.784 |  | 203 | 1.16 (0.95–1.43) | 0.153 | 0.0 | 3.2 | 0.531 |  | 395 | 1.14 (0.98–1.32) | 0.088 | 0.0 | 2.3 | 0.689 |
|  | Highest fourth | 595 | 1.16 (1.02–1.31) | 0.020 | 0.0 | 3.2 | 0.526 |  | 207 | 1.20 (0.97–1.48) | 0.087 | 25.2 | 5.3 | 0.254 |  | 388 | 1.13 (0.97–1.32) | 0.105 | 0.0 | 1.6 | 0.800 |
|  | *Linear trend (per 100g)* | *2277* | *1.03 (0.99–1.06)* | *0.098* | *0.0* | *2.8* | *0.590* |  | *771* | *1.03 (0.98–1.09)* | *0.276* | *0.0* | *2.0* | *0.744* |  | *1506* | *1.03 (0.99–1.07)* | *0.194* | *0.0* | *1.8* | *0.780* |
|  |  |  |  |  |  |  |  |  |  |  |  |  |  |  |  |  |  |  |  |  |  |
| **Nuts** | Lowest fourth | 701 | 1.00 (reference) | n/a | n/a | n/a | n/a |  | 236 | 1.00 (reference) | n/a | n/a | n/a | n/a |  | 465 | 1.00 (reference) | n/a | n/a | n/a | n/a |
|  | Second fourth | 468 | 1.14 (1.01–1.29) | 0.034 | 0.0 | 3.7 | 0.449 |  | 160 | 1.08 (0.88–1.34) | 0.449 | 25.0 | 5.3 | 0.255 |  | 308 | 1.18 (1.01–1.37) | 0.034 | 40.6 | 6.7 | 0.151 |
|  | Third fourth | 387 | 0.99 (0.87–1.13) | 0.884 | 0.0 | 1.6 | 0.809 |  | 135 | 0.96 (0.76–1.20) | 0.702 | 44.4 | 7.2 | 0.126 |  | 252 | 1.01 (0.86–1.20) | 0.879 | 61.7 | 10.5 | 0.033 |
|  | Highest fourth | 403 | 1.08 (0.94–1.23) | 0.279 | 0.0 | 2.3 | 0.680 |  | 149 | 1.09 (0.88–1.36) | 0.418 | 0.0 | 3.6 | 0.458 |  | 254 | 1.07 (0.91–1.27) | 0.416 | 29.7 | 5.7 | 0.224 |
|  | *Linear trend (per 10g)* | *1959* | *1.03 (0.97–1.08)* | *0.353* | *0.0* | *2.6* | *0.619* |  | *680* | *1.04 (0.95–1.13)* | *0.397* | *0.0* | *2.4* | *0.659* |  | *1279* | *1.02 (0.95–1.09)* | *0.549* | *0.0* | *2.6* | *0.635* |
|  |  |  |  |  |  |  |  |  |  |  |  |  |  |  |  |  |  |  |  |  |  |
| **Grains/ cereal** | Lowest fourth | 543 | 1.00 (reference) | n/a | n/a | n/a | n/a |  | 180 | 1.00 (reference) | n/a | n/a | n/a | n/a |  | 363 | 1.00 (reference) | n/a | n/a | n/a | n/a |
|  | Second fourth | 566 | 1.01 (0.90–1.14) | 0.875 | 0.0 | 1.4 | 0.847 |  | 189 | 1.00 (0.81–1.23) | 0.991 | 0.0 | 3.9 | 0.419 |  | 377 | 1.01 (0.88–1.17) | 0.864 | 0.0 | 2.8 | 0.593 |
|  | Third fourth | 572 | 1.00 (0.89–1.13) | 0.972 | 37.9 | 6.4 | 0.168 |  | 193 | 1.00 (0.82–1.24) | 0.965 | 0.0 | 2.5 | 0.645 |  | 379 | 1.00 (0.86–1.16) | 0.997 | 21.6 | 5.1 | 0.277 |
|  | Highest fourth | 617 | 1.08 (0.96–1.22) | 0.204 | 38.6 | 6.5 | 0.164 |  | 215 | 1.10 (0.89–1.35) | 0.365 | 55.2 | 8.9 | 0.063 |  | 402 | 1.07 (0.92–1.24) | 0.369 | 0.0 | 1.9 | 0.763 |
|  | *Linear trend (per 50g)* | *2298* | *1.03 (0.98–1.08)* | *0.205* | *0.0* | *0.7* | *0.947* |  | *777* | *1.08 (1.01–1.16)* | *0.029* | *0.0* | *3.1* | *0.538* |  | *1521* | *1.00 (0.94–1.07)* | *0.978* | *0.0* | *0.8* | *0.932* |
|  |  |  |  |  |  |  |  |  |  |  |  |  |  |  |  |  |  |  |  |  |  |
| **Red meat** | Lowest fourth | 573 | 1.00 (reference) | n/a | n/a | n/a | n/a |  | 200 | 1.00 (reference) | n/a | n/a | n/a | n/a |  | 373 | 1.00 (reference) | n/a | n/a | n/a | n/a |
|  | Second fourth | 607 | 1.05 (0.93–1.18) | 0.426 | 56.5 | 9.2 | 0.056 |  | 210 | 1.02 (0.84–1.24) | 0.849 | 30.0 | 5.7 | 0.221 |  | 397 | 1.06 (0.92–1.23) | 0.401 | 15.0 | 4.7 | 0.319 |
|  | Third fourth | 533 | 0.93 (0.82–1.05) | 0.223 | 28.0 | 5.6 | 0.235 |  | 190 | 0.93 (0.76–1.14) | 0.501 | 0.0 | 3.7 | 0.446 |  | 343 | 0.92 (0.80–1.07) | 0.297 | 0.0 | 2.5 | 0.644 |
|  | Highest fourth | 530 | 0.94 (0.83–1.06) | 0.307 | 28.5 | 5.6 | 0.232 |  | 161 | 0.81 (0.66–1.01) | 0.058 | 0.0 | 0.6 | 0.963 |  | 369 | 1.00 (0.86–1.16) | 0.968 | 65.4 | 11.6 | 0.021 |
|  | *Linear trend (per 50g)* | *2243* | *0.94 (0.87–1.02)* | *0.133* | *0.0* | *3.4* | *0.487* |  | *761* | *0.86 (0.75–0.98)* | *0.026* | *0.0* | *1.8* | *0.766* |  | *1482* | *1.01 (0.92–1.10)* | *0.908* | *74.8* | *15.9* | *0.003* |
|  |  |  |  |  |  |  |  |  |  |  |  |  |  |  |  |  |  |  |  |  |  |
| **Processed meat** | Lowest fourth | 657 | 1.00 (reference) | n/a | n/a | n/a | n/a |  | 239 | 1.00 (reference) | n/a | n/a | n/a | n/a |  | 418 | 1.00 (reference) | n/a | n/a | n/a | n/a |
|  | Second fourth | 500 | 1.03 (0.91–1.16) | 0.644 | 0.0 | 1.5 | 0.831 |  | 159 | 0.87 (0.71–1.07) | 0.178 | 0.0 | 3.4 | 0.498 |  | 341 | 1.12 (0.97–1.30) | 0.122 | 0.0 | 3.3 | 0.514 |
|  | Third fourth | 582 | 1.01 (0.90–1.13) | 0.835 | 0.0 | 2.0 | 0.728 |  | 193 | 0.90 (0.74–1.09) | 0.286 | 0.0 | 3.5 | 0.483 |  | 389 | 1.08 (0.93–1.24) | 0.306 | 0.0 | 1.7 | 0.783 |
|  | Highest fourth | 504 | 0.89 (0.79–1.00) | 0.057 | 0.0 | 2.8 | 0.592 |  | 170 | 0.80 (0.65–0.98) | 0.030 | 0.0 | 2.7 | 0.617 |  | 334 | 0.95 (0.82–1.09) | 0.453 | 33.0 | 6.0 | 0.202 |
|  | *Linear trend (per 10g)* | *2243* | *0.98 (0.95–1.01)* | *0.216* | *0.0* | *2.2* | *0.706* |  | *761* | *0.97 (0.92–1.03)* | *0.339* | *24.3* | *5.3* | *0.260* |  | *1482* | *0.99 (0.95–1.03)* | *0.538* | *0.0* | *3.7* | *0.444* |
|  |  |  |  |  |  |  |  |  |  |  |  |  |  |  |  |  |  |  |  |  |  |
| **White meat** | Lowest fourth | 552 | 1.00 (reference) | n/a | n/a | n/a | n/a |  | 195 | 1.00 (reference) | n/a | n/a | n/a | n/a |  | 357 | 1.00 (reference) | n/a | n/a | n/a | n/a |
|  | Second fourth | 594 | 1.06 (0.94–1.19) | 0.326 | 0.0 | 4.0 | 0.406 |  | 200 | 1.01 (0.83–1.23) | 0.909 | 0.0 | 1.8 | 0.775 |  | 394 | 1.09 (0.94–1.26) | 0.260 | 0.0 | 3.0 | 0.553 |
|  | Third fourth | 564 | 1.01 (0.89–1.13) | 0.916 | 42.6 | 7.0 | 0.137 |  | 176 | 0.91 (0.74–1.12) | 0.376 | 0.0 | 1.8 | 0.773 |  | 388 | 1.06 (0.91–1.22) | 0.454 | 34.0 | 6.1 | 0.195 |
|  | Highest fourth | 533 | 0.96 (0.85–1.09) | 0.529 | 0.0 | 1.6 | 0.816 |  | 190 | 1.01 (0.82–1.25) | 0.906 | 21.2 | 5.1 | 0.280 |  | 343 | 0.94 (0.80–1.09) | 0.400 | 0.0 | 3.0 | 0.550 |
|  | *Linear trend (per 50g)* | *2243* | *0.97 (0.91–1.04)* | *0.375* | *0.0* | *0.7* | *0.947* |  | *761* | *1.01 (0.91–1.12)* | *0.884* | *42.4* | *6.9* | *0.139* |  | *1482* | *0.96 (0.89–1.05)* | *0.382* | *0.0* | *2.5* | *0.641* |
|  |  |  |  |  |  |  |  |  |  |  |  |  |  |  |  |  |  |  |  |  |  |
| **Fish** | Lowest fourth | 542 | 1.00 (reference) | n/a | n/a | n/a | n/a |  | 195 | 1.00 (reference) | n/a | n/a | n/a | n/a |  | 347 | 1.00 (reference) | n/a | n/a | n/a | n/a |
|  | Second fourth | 589 | 1.07 (0.95–1.21) | 0.256 | 58.4 | 9.6 | 0.047 |  | 205 | 1.04 (0.86–1.27) | 0.673 | 25.3 | 5.4 | 0.253 |  | 384 | 1.09 (0.94–1.26) | 0.268 | 42.1 | 6.9 | 0.141 |
|  | Third fourth | 573 | 1.03 (0.92–1.16) | 0.614 | 69.8 | 13.2 | 0.010 |  | 184 | 0.93 (0.76–1.14) | 0.492 | 10.5 | 4.5 | 0.346 |  | 389 | 1.08 (0.94–1.26) | 0.278 | 63.9 | 11.1 | 0.026 |
|  | Highest fourth | 542 | 0.98 (0.87–1.11) | 0.771 | 10.1 | 4.4 | 0.349 |  | 186 | 0.95 (0.77–1.16) | 0.607 | 0.0 | 1.6 | 0.814 |  | 356 | 1.00 (0.86–1.16) | 0.981 | 28.4 | 5.6 | 0.232 |
|  | *Linear trend (per 50g)* | *2246* | *0.97 (0.84–1.12)* | *0.701* | *12.8* | *4.6* | *0.332* |  | *770* | *1.00 (0.80–1.25)* | *0.991* | *26.4* | *5.4* | *0.245* |  | *1476* | *0.98 (0.82–1.17)* | *0.847* | *9.6* | *4.4* | *0.351* |
|  |  |  |  |  |  |  |  |  |  |  |  |  |  |  |  |  |  |  |  |  |  |
| **Eggs** | Lowest fourth | 567 | 1.00 (reference) | n/a | n/a | n/a | n/a |  | 199 | 1.00 (reference) | n/a | n/a | n/a | n/a |  | 368 | 1.00 (reference) | n/a | n/a | n/a | n/a |
|  | Second fourth | 535 | 0.93 (0.83–1.05) | 0.256 | 0.0 | 1.1 | 0.901 |  | 187 | 0.92 (0.75–1.13) | 0.446 | 6.0 | 4.3 | 0.372 |  | 348 | 0.94 (0.81–1.09) | 0.411 | 0.0 | 2.8 | 0.600 |
|  | Third fourth | 613 | 1.07 (0.95–1.20) | 0.263 | 0.0 | 2.3 | 0.687 |  | 197 | 0.99 (0.81–1.21) | 0.918 | 32.6 | 5.9 | 0.204 |  | 416 | 1.11 (0.97–1.28) | 0.141 | 0.0 | 2.3 | 0.686 |
|  | Highest fourth | 575 | 1.04 (0.92–1.17) | 0.547 | 14.2 | 4.7 | 0.324 |  | 192 | 0.98 (0.80–1.20) | 0.857 | 31.7 | 5.9 | 0.210 |  | 383 | 1.07 (0.92–1.24) | 0.368 | 0.0 | 1.3 | 0.869 |
|  | *Linear trend (per 10g)* | *2290* | *0.99 (0.95–1.03)* | *0.762* | *51.3* | *8.2* | *0.084* |  | *775* | *0.98 (0.91–1.05)* | *0.507* | *43.4* | *7.1* | *0.132* |  | *1515* | *1.01 (0.96–1.06)* | *0.783* | *0.0* | *3.1* | *0.542* |
|  |  |  |  |  |  |  |  |  |  |  |  |  |  |  |  |  |  |  |  |  |  |
| **Dairy products** | Lowest fourth | 552 | 1.00 (reference) | n/a | n/a | n/a | n/a |  | 192 | 1.00 (reference) | n/a | n/a | n/a | n/a |  | 360 | 1.00 (reference) | n/a | n/a | n/a | n/a |
|  | Second fourth | 587 | 1.01 (0.90–1.14) | 0.819 | 54.5 | 8.8 | 0.067 |  | 207 | 1.03 (0.84–1.26) | 0.765 | 25.9 | 5.4 | 0.249 |  | 380 | 1.00 (0.87–1.16) | 0.987 | 33.4 | 6.0 | 0.198 |
|  | Third fourth | 602 | 1.04 (0.92–1.16) | 0.558 | 0.0 | 2.2 | 0.700 |  | 202 | 1.00 (0.82–1.23) | 0.979 | 0.0 | 3.6 | 0.466 |  | 400 | 1.05 (0.91–1.21) | 0.512 | 0.0 | 2.2 | 0.700 |
|  | Highest fourth | 572 | 0.99 (0.87–1.11) | 0.822 | 0.0 | 1.6 | 0.812 |  | 181 | 0.91 (0.73–1.12) | 0.358 | 19.3 | 5.0 | 0.292 |  | 391 | 1.02 (0.88–1.19) | 0.746 | 0.0 | 3.6 | 0.459 |
|  | *Linear trend (per 100g)* | *2313* | *1.00 (0.98–1.02)* | *0.959* | *3.5* | *4.1* | *0.386* |  | *782* | *1.01 (0.98–1.05)* | *0.409* | *0.0* | *1.8* | *0.772* |  | *1531* | *1.00 (0.97–1.02)* | *0.748* | *62.5* | *10.7* | *0.030* |
|  |  |  |  |  |  |  |  |  |  |  |  |  |  |  |  |  |  |  |  |  |  |
| **Cheese** | Lowest fourth | 546 | 1.00 (reference) | n/a | n/a | n/a | n/a |  | 184 | 1.00 (reference) | n/a | n/a | n/a | n/a |  | 362 | 1.00 (reference) | n/a | n/a | n/a | n/a |
|  | Second fourth | 536 | 0.97 (0.86–1.09) | 0.583 | 0.0 | 1.7 | 0.783 |  | 178 | 0.94 (0.76–1.16) | 0.571 | 0.0 | 2.4 | 0.660 |  | 358 | 0.98 (0.85–1.14) | 0.791 | 0.0 | 2.4 | 0.657 |
|  | Third fourth | 534 | 0.96 (0.85–1.09) | 0.541 | 0.0 | 1.1 | 0.890 |  | 179 | 0.96 (0.78–1.19) | 0.725 | 0.0 | 1.6 | 0.800 |  | 355 | 0.96 (0.83–1.12) | 0.637 | 13.6 | 4.6 | 0.327 |
|  | Highest fourth | 591 | 1.07 (0.95–1.20) | 0.264 | 0.0 | 3.3 | 0.505 |  | 210 | 1.14 (0.93–1.40) | 0.216 | 60.2 | 10.1 | 0.039 |  | 381 | 1.04 (0.89–1.20) | 0.643 | 0.0 | 2.5 | 0.638 |
|  | *Linear trend (per 10g)* | *2207* | *1.03 (0.96–1.11)* | *0.373* | *32.5* | *5.9* | *0.204* |  | *751* | *1.05 (0.94–1.18)* | *0.367* | *68.6* | *12.7* | *0.013* |  | *1456* | *1.02 (0.94–1.11)* | *0.602* | *0.0* | *1.6* | *0.816* |
|  |  |  |  |  |  |  |  |  |  |  |  |  |  |  |  |  |  |  |  |  |  |
| **Coffee** | Lowest fourth | 529 | 1.00 (reference) | n/a | n/a | n/a | n/a |  | 182 | 1.00 (reference) | n/a | n/a | n/a | n/a |  | 347 | 1.00 (reference) | n/a | n/a | n/a | n/a |
|  | Second fourth | 539 | 1.02 (0.90–1.15) | 0.795 | 0.0 | 1.2 | 0.877 |  | 192 | 1.10 (0.89–1.35) | 0.386 | 0.0 | 1.9 | 0.749 |  | 347 | 0.97 (0.84–1.14) | 0.740 | 0.0 | 3.0 | 0.563 |
|  | Third fourth | 549 | 1.03 (0.91–1.17) | 0.644 | 47.9 | 7.7 | 0.104 |  | 190 | 1.09 (0.88–1.35) | 0.436 | 41.9 | 6.9 | 0.142 |  | 359 | 1.00 (0.86–1.17) | 0.977 | 0.0 | 3.0 | 0.553 |
|  | Highest fourth | 517 | 1.01 (0.89–1.16) | 0.840 | 0.0 | 3.3 | 0.514 |  | 163 | 0.97 (0.77–1.21) | 0.771 | 0.0 | 1.9 | 0.758 |  | 354 | 1.04 (0.89–1.22) | 0.617 | 0.0 | 1.5 | 0.831 |
|  | *Linear trend (per 100g)* | *2134* | *1.00 (0.99–1.01)* | *0.560* | *0.0* | *3.4* | *0.490* |  | *727* | *0.99 (0.98–1.00)* | *0.169* | *0.0* | *2.1* | *0.712* |  | *1407* | *1.01 (1.00–1.02)* | *0.067* | *0.0* | *1.0* | *0.914* |
|  |  |  |  |  |  |  |  |  |  |  |  |  |  |  |  |  |  |  |  |  |  |
| **Tea** | Lowest fourth | 542 | 1.00 (reference) | n/a | n/a | n/a | n/a |  | 179 | 1.00 (reference) | n/a | n/a | n/a | n/a |  | 363 | 1.00 (reference) | n/a | n/a | n/a | n/a |
|  | Second fourth | 557 | 1.00 (0.89–1.13) | 0.992 | 25.1 | 5.3 | 0.254 |  | 201 | 1.13 (0.92–1.39) | 0.227 | 0.0 | 2.6 | 0.632 |  | 356 | 0.94 (0.81–1.08) | 0.376 | 0.0 | 3.4 | 0.497 |
|  | Third fourth | 545 | 0.97 (0.86–1.09) | 0.620 | 0.0 | 1.4 | 0.842 |  | 184 | 1.04 (0.84–1.28) | 0.723 | 0.0 | 3.3 | 0.503 |  | 361 | 0.94 (0.81–1.09) | 0.390 | 24.3 | 5.3 | 0.259 |
|  | Highest fourth | 563 | 1.01 (0.89–1.14) | 0.925 | 0.0 | 1.6 | 0.812 |  | 176 | 1.02 (0.82–1.27) | 0.863 | 0.0 | 1.8 | 0.772 |  | 387 | 1.00 (0.86–1.16) | 0.955 | 0.0 | 2.0 | 0.739 |
|  | *Linear trend (per 100g)* | *2207* | *1.00 (0.99–1.01)* | *0.866* | *0.0* | *1.9* | *0.753* |  | *740* | *1.01 (0.99–1.03)* | *0.450* | *0.0* | *2.2* | *0.691* |  | *1467* | *1.00 (0.98–1.01)* | *0.845* | *0.0* | *2.4* | *0.658* |
|  |  |  |  |  |  |  |  |  |  |  |  |  |  |  |  |  |  |  |  |  |  |
| **Carbo-hydrate** | Lowest fourth | 515 | 1.00 (reference) | n/a | n/a | n/a | n/a |  | 173 | 1.00 (reference) | n/a | n/a | n/a | n/a |  | 342 | 1.00 (reference) | n/a | n/a | n/a | n/a |
|  | Second fourth | 581 | 1.10 (0.97–1.24) | 0.130 | 0.0 | 1.8 | 0.769 |  | 185 | 1.06 (0.86–1.31) | 0.573 | 0.0 | 1.2 | 0.882 |  | 396 | 1.12 (0.96–1.29) | 0.148 | 6.8 | 4.3 | 0.368 |
|  | Third fourth | 604 | 1.13 (1.00–1.28) | 0.049 | 28.8 | 5.6 | 0.229 |  | 223 | 1.28 (1.04–1.58) | 0.020 | 3.6 | 4.1 | 0.386 |  | 381 | 1.06 (0.91–1.23) | 0.482 | 41.9 | 6.9 | 0.142 |
|  | Highest fourth | 613 | 1.17 (1.03–1.33) | 0.018 | 0.0 | 1.5 | 0.832 |  | 201 | 1.18 (0.94–1.48) | 0.148 | 48.9 | 7.8 | 0.098 |  | 412 | 1.15 (0.99–1.35) | 0.073 | 28.3 | 5.6 | 0.233 |
|  | *Linear trend (per 1% energy)* | *2313* | *1.01 (1.00–1.01)* | *0.033* | *0.0* | *2.1* | *0.719* |  | *782* | *1.01 (1.00–1.02)* | *0.072* | *18.0* | *4.9* | *0.300* |  | *1531* | *1.01 (1.00–1.01)* | *0.190* | *25.3* | *5.4* | *0.253* |
|  |  |  |  |  |  |  |  |  |  |  |  |  |  |  |  |  |  |  |  |  |  |
| **Protein** | Lowest fourth | 588 | 1.00 (reference) | n/a | n/a | n/a | n/a |  | 207 | 1.00 (reference) | n/a | n/a | n/a | n/a |  | 381 | 1.00 (reference) | n/a | n/a | n/a | n/a |
|  | Second fourth | 585 | 0.97 (0.86–1.08) | 0.550 | 0.0 | 1.6 | 0.817 |  | 215 | 1.00 (0.83–1.21) | 0.986 | 0.0 | 1.5 | 0.830 |  | 370 | 0.94 (0.82–1.09) | 0.431 | 0.0 | 2.6 | 0.633 |
|  | Third fourth | 598 | 0.98 (0.87–1.10) | 0.757 | 0.0 | 1.5 | 0.823 |  | 193 | 0.90 (0.74–1.10) | 0.306 | 0.0 | 3.2 | 0.525 |  | 405 | 1.03 (0.89–1.18) | 0.726 | 0.0 | 3.5 | 0.475 |
|  | Highest fourth | 542 | 0.90 (0.80–1.01) | 0.079 | 0.0 | 2.4 | 0.668 |  | 167 | 0.80 (0.64–0.98) | 0.035 | 39.5 | 6.6 | 0.158 |  | 375 | 0.95 (0.82–1.10) | 0.515 | 0.0 | 1.4 | 0.848 |
|  | *Linear trend (per 1% energy)* | *2313* | *0.99 (0.97–1.01)* | *0.301* | *0.0* | *2.8* | *0.592* |  | *782* | *0.98 (0.95–1.01)* | *0.228* | *45.9* | *7.4* | *0.117* |  | *1531* | *1.00 (0.97–1.02)* | *0.725* | *0.0* | *1.1* | *0.902* |
|  |  |  |  |  |  |  |  |  |  |  |  |  |  |  |  |  |  |  |  |  |  |
| **Total fat** | Lowest fourth | 590 | 1.00 (reference) | n/a | n/a | n/a | n/a |  | 192 | 1.00 (reference) | n/a | n/a | n/a | n/a |  | 398 | 1.00 (reference) | n/a | n/a | n/a | n/a |
|  | Second fourth | 584 | 0.98 (0.87–1.10) | 0.689 | 0.0 | 0.3 | 0.990 |  | 203 | 1.02 (0.84–1.25) | 0.827 | 0.0 | 0.4 | 0.986 |  | 381 | 0.96 (0.83–1.10) | 0.527 | 0.0 | 0.8 | 0.937 |
|  | Third fourth | 565 | 0.95 (0.84–1.07) | 0.388 | 1.3 | 4.1 | 0.399 |  | 185 | 0.93 (0.75–1.14) | 0.469 | 10.5 | 4.5 | 0.346 |  | 380 | 0.96 (0.83–1.11) | 0.592 | 0.0 | 3.2 | 0.523 |
|  | Highest fourth | 574 | 0.98 (0.87–1.11) | 0.777 | 0.0 | 2.9 | 0.576 |  | 202 | 1.01 (0.82–1.25) | 0.906 | 0.0 | 3.5 | 0.481 |  | 372 | 0.97 (0.84–1.12) | 0.675 | 5.0 | 4.2 | 0.378 |
|  | *Linear trend (per 1% energy)* | *2313* | *1.00 (0.99–1.01)* | *0.661* | *0.0* | *1.2* | *0.870* |  | *782* | *1.00 (0.98–1.01)* | *0.493* | *7.3* | *4.3* | *0.365* |  | *1531* | *1.00 (0.99–1.01)* | *0.969* | *0.0* | *2.2* | *0.708* |
|  |  |  |  |  |  |  |  |  |  |  |  |  |  |  |  |  |  |  |  |  |  |
| **Saturated fat** | Lowest fourth | 572 | 1.00 (reference) | n/a | n/a | n/a | n/a |  | 195 | 1.00 (reference) | n/a | n/a | n/a | n/a |  | 377 | 1.00 (reference) | n/a | n/a | n/a | n/a |
|  | Second fourth | 588 | 1.02 (0.91–1.15) | 0.739 | 0.0 | 1.3 | 0.862 |  | 197 | 0.98 (0.81–1.20) | 0.878 | 0.0 | 2.2 | 0.701 |  | 391 | 1.04 (0.90–1.20) | 0.598 | 0.0 | 0.4 | 0.984 |
|  | Third fourth | 576 | 1.00 (0.89–1.13) | 0.975 | 0.0 | 3.9 | 0.424 |  | 188 | 0.94 (0.76–1.15) | 0.528 | 37.9 | 6.4 | 0.169 |  | 388 | 1.04 (0.90–1.20) | 0.612 | 0.0 | 3.5 | 0.485 |
|  | Highest fourth | 577 | 1.02 (0.90–1.15) | 0.789 | 9.3 | 4.4 | 0.353 |  | 202 | 1.01 (0.82–1.25) | 0.914 | 0.0 | 3.4 | 0.493 |  | 375 | 1.02 (0.88–1.19) | 0.802 | 15.2 | 4.7 | 0.317 |
|  | *Linear trend (per 1% energy)* | *2313* | *1.00 (0.98–1.02)* | *0.709* | *0.0* | *3.3* | *0.515* |  | *782* | *0.99 (0.96–1.02)* | *0.483* | *27.0* | *5.5* | *0.241* |  | *1531* | *1.00 (0.98–1.03)* | *0.943* | *0.0* | *3.9* | *0.423* |
|  |  |  |  |  |  |  |  |  |  |  |  |  |  |  |  |  |  |  |  |  |  |
| **Mono-unsaturated fat** | Lowest fourth | 595 | 1.00 (reference) | n/a | n/a | n/a | n/a |  | 188 | 1.00 (reference) | n/a | n/a | n/a | n/a |  | 407 | 1.00 (reference) | n/a | n/a | n/a | n/a |
|  | Second fourth | 589 | 0.98 (0.87–1.10) | 0.728 | 0.0 | 0.9 | 0.929 |  | 203 | 1.05 (0.86–1.28) | 0.627 | 0.0 | 1.2 | 0.872 |  | 386 | 0.95 (0.82–1.09) | 0.450 | 0.0 | 0.4 | 0.986 |
|  | Third fourth | 577 | 0.96 (0.86–1.08) | 0.522 | 0.0 | 1.7 | 0.798 |  | 200 | 1.03 (0.84–1.27) | 0.763 | 13.0 | 4.6 | 0.331 |  | 377 | 0.93 (0.81–1.07) | 0.328 | 0.0 | 3.6 | 0.457 |
|  | Highest fourth | 552 | 0.94 (0.83–1.06) | 0.328 | 0.0 | 0.3 | 0.990 |  | 191 | 0.99 (0.80–1.22) | 0.903 | 0.0 | 3.8 | 0.434 |  | 361 | 0.92 (0.79–1.07) | 0.273 | 0.0 | 2.8 | 0.590 |
|  | *Linear trend (per 1% energy)* | *2313* | *0.99 (0.97–1.01)* | *0.496* | *0.0* | *1.0* | *0.909* |  | *782* | *0.98 (0.95–1.02)* | *0.311* | *0.0* | *4.0* | *0.410* |  | *1531* | *1.00 (0.97–1.02)* | *0.944* | *0.0* | *3.9* | *0.425* |
|  |  |  |  |  |  |  |  |  |  |  |  |  |  |  |  |  |  |  |  |  |  |
| **Poly-unsaturated fat** | Lowest fourth | 562 | 1.00 (reference) | n/a | n/a | n/a | n/a |  | 188 | 1.00 (reference) | n/a | n/a | n/a | n/a |  | 374 | 1.00 (reference) | n/a | n/a | n/a | n/a |
|  | Second fourth | 615 | 1.07 (0.96–1.21) | 0.220 | 31.1 | 5.8 | 0.214 |  | 192 | 0.99 (0.80–1.21) | 0.886 | 44.9 | 7.3 | 0.123 |  | 423 | 1.12 (0.97–1.29) | 0.122 | 5.4 | 4.2 | 0.376 |
|  | Third fourth | 561 | 0.98 (0.87–1.11) | 0.776 | 23.7 | 5.2 | 0.263 |  | 197 | 1.02 (0.83–1.25) | 0.875 | 4.6 | 4.2 | 0.381 |  | 364 | 0.96 (0.83–1.12) | 0.631 | 0.0 | 2.2 | 0.707 |
|  | Highest fourth | 575 | 1.02 (0.91–1.15) | 0.748 | 0.0 | 2.9 | 0.574 |  | 205 | 1.07 (0.87–1.31) | 0.521 | 35.9 | 6.2 | 0.182 |  | 370 | 1.00 (0.86–1.15) | 0.959 | 0.0 | 1.0 | 0.913 |
|  | *Linear trend (per 1% energy)* | *2313* | *1.00 (0.98–1.03)* | *0.835* | *0.0* | *3.2* | *0.523* |  | *782* | *1.02 (0.98–1.07)* | *0.386* | *37.9* | *6.4* | *0.169* |  | *1531* | *0.99 (0.96–1.03)* | *0.743* | *0.0* | *0.7* | *0.951* |
|  |  |  |  |  |  |  |  |  |  |  |  |  |  |  |  |  |  |  |  |  |  |
| **Alcohol** | Lowest fourth | 791 | 1.00 (reference) | n/a | n/a | n/a | n/a |  | 274 | 1.00 (reference) | n/a | n/a | n/a | n/a |  | 517 | 1.00 (reference) | n/a | n/a | n/a | n/a |
|  | Second fourth | 456 | 1.00 (0.89–1.13) | 0.971 | 18.1 | 4.9 | 0.299 |  | 159 | 0.96 (0.78–1.19) | 0.728 | 0.0 | 2.3 | 0.672 |  | 297 | 1.02 (0.88–1.19) | 0.762 | 44.7 | 7.2 | 0.124 |
|  | Third fourth | 554 | 1.05 (0.94–1.18) | 0.386 | 0.0 | 0.7 | 0.947 |  | 171 | 0.91 (0.75–1.12) | 0.382 | 0.0 | 2.2 | 0.694 |  | 383 | 1.13 (0.98–1.29) | 0.093 | 0.0 | 0.3 | 0.987 |
|  | Highest fourth | 512 | 1.02 (0.91–1.15) | 0.723 | 0.0 | 1.5 | 0.828 |  | 178 | 1.00 (0.82–1.22) | 0.994 | 0.0 | 2.0 | 0.736 |  | 334 | 1.03 (0.89–1.20) | 0.658 | 0.0 | 2.2 | 0.694 |
|  | *Linear trend (per 10g)* | *2313* | *0.97 (0.94–1.01)* | *0.117* | *11.8* | *4.5* | *0.338* |  | *782* | *0.98 (0.93–1.03)* | *0.404* | *0.0* | *1.8* | *0.781* |  | *1531* | *0.97 (0.94–1.01)* | *0.186* | *0.0* | *3.1* | *0.542* |
|  |  |  |  |  |  |  |  |  |  |  |  |  |  |  |  |  |  |  |  |  |  |
| **Fibre** | Lowest fourth | 513 | 1.00 (reference) | n/a | n/a | n/a | n/a |  | 160 | 1.00 (reference) | n/a | n/a | n/a | n/a |  | 353 | 1.00 (reference) | n/a | n/a | n/a | n/a |
|  | Second fourth | 566 | 1.06 (0.94–1.20) | 0.319 | 0.0 | 3.1 | 0.540 |  | 196 | 1.17 (0.95–1.45) | 0.137 | 0.0 | 2.9 | 0.582 |  | 370 | 1.01 (0.87–1.17) | 0.869 | 0.0 | 3.3 | 0.505 |
|  | Third fourth | 605 | 1.12 (1.00–1.27) | 0.060 | 50.7 | 8.1 | 0.088 |  | 202 | 1.19 (0.96–1.48) | 0.108 | 0.0 | 2.8 | 0.598 |  | 403 | 1.09 (0.94–1.27) | 0.234 | 48.8 | 7.8 | 0.099 |
|  | Highest fourth | 629 | 1.17 (1.04–1.33) | 0.011 | 0.0 | 3.9 | 0.423 |  | 224 | 1.33 (1.08–1.66) | 0.009 | 0.0 | 3.9 | 0.422 |  | 405 | 1.10 (0.95–1.28) | 0.210 | 37.1 | 6.4 | 0.174 |
|  | *Linear trend (per 5g)* | *2313* | *1.08 (1.02–1.15)* | *0.008* | *0.0* | *2.3* | *0.680* |  | *782* | *1.13 (1.02–1.24)* | *0.015* | *0.0* | *1.3* | *0.863* |  | *1531* | *1.06 (0.98–1.14)* | *0.121* | *2.9* | *4.1* | *0.390* |
|  |  |  |  |  |  |  |  |  |  |  |  |  |  |  |  |  |  |  |  |  |  |
| **Carotene** | Lowest fourth | 523 | 1.00 (reference) | n/a | n/a | n/a | n/a |  | 166 | 1.00 (reference) | n/a | n/a | n/a | n/a |  | 357 | 1.00 (reference) | n/a | n/a | n/a | n/a |
|  | Second fourth | 623 | 1.16 (1.03–1.31) | 0.013 | 39.1 | 6.6 | 0.160 |  | 192 | 1.10 (0.89–1.36) | 0.372 | 0.0 | 3.9 | 0.419 |  | 431 | 1.19 (1.03–1.37) | 0.018 | 44.2 | 7.2 | 0.127 |
|  | Third fourth | 549 | 1.02 (0.91–1.16) | 0.707 | 45.5 | 7.3 | 0.119 |  | 189 | 1.07 (0.86–1.33) | 0.532 | 0.0 | 3.3 | 0.508 |  | 360 | 1.00 (0.86–1.16) | 0.999 | 22.7 | 5.2 | 0.270 |
|  | Highest fourth | 618 | 1.16 (1.03–1.31) | 0.013 | 0.0 | 1.8 | 0.773 |  | 235 | 1.34 (1.09–1.65) | 0.005 | 0.0 | 3.2 | 0.527 |  | 383 | 1.08 (0.93–1.25) | 0.330 | 0.0 | 2.9 | 0.566 |
|  | *Linear trend (per 1000ug)* | *2313* | *1.03 (0.96–1.09)* | *0.415* | *0.0* | *3.0* | *0.559* |  | *782* | *1.09 (0.99–1.21)* | *0.094* | *31.0* | *5.8* | *0.215* |  | *1531* | *1.00 (0.92–1.08)* | *0.982* | *0.0* | *2.3* | *0.685* |
|  |  |  |  |  |  |  |  |  |  |  |  |  |  |  |  |  |  |  |  |  |  |
| **Vitamin C** | Lowest fourth | 549 | 1.00 (reference) | n/a | n/a | n/a | n/a |  | 184 | 1.00 (reference) | n/a | n/a | n/a | n/a |  | 365 | 1.00 (reference) | n/a | n/a | n/a | n/a |
|  | Second fourth | 541 | 0.95 (0.84–1.07) | 0.412 | 17.5 | 4.8 | 0.303 |  | 180 | 0.93 (0.76–1.15) | 0.516 | 0.0 | 1.8 | 0.780 |  | 361 | 0.96 (0.83–1.12) | 0.623 | 37.3 | 6.4 | 0.173 |
|  | Third fourth | 626 | 1.09 (0.97–1.23) | 0.141 | 0.0 | 0.3 | 0.990 |  | 214 | 1.09 (0.89–1.33) | 0.415 | 0.0 | 1.7 | 0.794 |  | 412 | 1.09 (0.95–1.26) | 0.230 | 0.0 | 2.3 | 0.674 |
|  | Highest fourth | 597 | 1.06 (0.94–1.19) | 0.347 | 0.0 | 0.7 | 0.956 |  | 204 | 1.06 (0.86–1.30) | 0.605 | 0.0 | 1.6 | 0.817 |  | 393 | 1.06 (0.92–1.23) | 0.432 | 0.0 | 3.1 | 0.544 |
|  | *Linear trend (per 50mg)* | *2313* | *1.02 (0.99–1.05)* | *0.282* | *0.0* | *1.3* | *0.864* |  | *782* | *1.01 (0.96–1.06)* | *0.702* | *0.0* | *3.0* | *0.556* |  | *1531* | *1.02 (0.99–1.06)* | *0.242* | *0.0* | *2.2* | *0.700* |
|  |  |  |  |  |  |  |  |  |  |  |  |  |  |  |  |  |  |  |  |  |  |
| **Vitamin E** | Lowest fourth | 555 | 1.00 (reference) | n/a | n/a | n/a | n/a |  | 179 | 1.00 (reference) | n/a | n/a | n/a | n/a |  | 376 | 1.00 (reference) | n/a | n/a | n/a | n/a |
|  | Second fourth | 579 | 1.02 (0.90–1.14) | 0.798 | 31.2 | 5.8 | 0.213 |  | 196 | 1.06 (0.86–1.30) | 0.607 | 57.1 | 9.3 | 0.053 |  | 383 | 0.99 (0.86–1.15) | 0.936 | 0.0 | 3.8 | 0.436 |
|  | Third fourth | 594 | 1.04 (0.93–1.17) | 0.503 | 0.0 | 3.5 | 0.475 |  | 209 | 1.12 (0.92–1.37) | 0.269 | 30.2 | 5.7 | 0.220 |  | 385 | 1.00 (0.87–1.16) | 0.986 | 0.0 | 1.9 | 0.751 |
|  | Highest fourth | 585 | 1.04 (0.92–1.16) | 0.564 | 0.0 | 1.6 | 0.817 |  | 198 | 1.07 (0.87–1.32) | 0.504 | 0.0 | 2.6 | 0.625 |  | 387 | 1.02 (0.88–1.18) | 0.815 | 0.0 | 3.0 | 0.566 |
|  | *Linear trend (per 5mg)* | *2313* | *1.01 (0.93–1.09)* | *0.872* | *0.0* | *1.2* | *0.884* |  | *782* | *0.97 (0.84–1.11)* | *0.664* | *0.0* | *0.8* | *0.943* |  | *1531* | *1.03 (0.94–1.13)* | *0.508* | *0.0* | *3.2* | *0.518* |
|  |  |  |  |  |  |  |  |  |  |  |  |  |  |  |  |  |  |  |  |  |  |
| **Folate** | Lowest fourth | 529 | 1.00 (reference) | n/a | n/a | n/a | n/a |  | 190 | 1.00 (reference) | n/a | n/a | n/a | n/a |  | 339 | 1.00 (reference) | n/a | n/a | n/a | n/a |
|  | Second fourth | 566 | 1.04 (0.92–1.17) | 0.569 | 0.0 | 1.3 | 0.867 |  | 193 | 0.98 (0.80–1.20) | 0.820 | 0.0 | 2.6 | 0.628 |  | 373 | 1.07 (0.92–1.24) | 0.390 | 0.0 | 2.5 | 0.651 |
|  | Third fourth | 597 | 1.08 (0.96–1.22) | 0.201 | 0.0 | 0.1 | 0.999 |  | 203 | 1.02 (0.83–1.25) | 0.849 | 0.0 | 2.4 | 0.658 |  | 394 | 1.11 (0.96–1.29) | 0.153 | 0.0 | 1.5 | 0.832 |
|  | Highest fourth | 621 | 1.13 (1.00–1.27) | 0.054 | 0.0 | 0.4 | 0.983 |  | 196 | 0.99 (0.80–1.22) | 0.924 | 0.0 | 3.1 | 0.547 |  | 425 | 1.21 (1.04–1.40) | 0.015 | 0.0 | 2.7 | 0.614 |
|  | *Linear trend (per 50ug)* | *2313* | *1.01 (0.99–1.03)* | *0.176* | *0.0* | *2.0* | *0.742* |  | *782* | *1.01 (0.98–1.04)* | *0.432* | *0.0* | *2.6* | *0.622* |  | *1531* | *1.01 (0.99–1.04)* | *0.211* | *48.2* | *7.7* | *0.102* |
|  |  |  |  |  |  |  |  |  |  |  |  |  |  |  |  |  |  |  |  |  |  |
| **Calcium** | Lowest fourth | 565 | 1.00 (reference) | n/a | n/a | n/a | n/a |  | 195 | 1.00 (reference) | n/a | n/a | n/a | n/a |  | 370 | 1.00 (reference) | n/a | n/a | n/a | n/a |
|  | Second fourth | 576 | 0.98 (0.87–1.10) | 0.687 | 1.4 | 4.1 | 0.398 |  | 198 | 0.97 (0.79–1.18) | 0.742 | 0.0 | 3.6 | 0.462 |  | 378 | 0.98 (0.85–1.13) | 0.758 | 58.5 | 9.6 | 0.047 |
|  | Third fourth | 576 | 0.97 (0.86–1.09) | 0.580 | 0.0 | 0.3 | 0.987 |  | 202 | 0.98 (0.80–1.20) | 0.830 | 0.0 | 2.8 | 0.584 |  | 374 | 0.96 (0.83–1.11) | 0.602 | 0.0 | 3.2 | 0.517 |
|  | Highest fourth | 596 | 1.00 (0.89–1.13) | 0.985 | 0.0 | 1.4 | 0.846 |  | 187 | 0.90 (0.73–1.11) | 0.321 | 19.2 | 4.9 | 0.293 |  | 409 | 1.06 (0.92–1.23) | 0.428 | 56.7 | 9.2 | 0.056 |
|  | *Linear trend (per 100mg)* | *2313* | *1.00 (0.98–1.02)* | *0.831* | *0.0* | *3.7* | *0.452* |  | *782* | *1.01 (0.98–1.04)* | *0.612* | *0.0* | *3.0* | *0.550* |  | *1531* | *1.00 (0.98–1.02)* | *0.915* | *65.7* | *11.7* | *0.020* |
|  |  |  |  |  |  |  |  |  |  |  |  |  |  |  |  |  |  |  |  |  |  |
| **Cholesterol** | Lowest fourth | 576 | 1.00 (reference) | n/a | n/a | n/a | n/a |  | 194 | 1.00 (reference) | n/a | n/a | n/a | n/a |  | 382 | 1.00 (reference) | n/a | n/a | n/a | n/a |
|  | Second fourth | 598 | 1.03 (0.92–1.15) | 0.642 | 0.0 | 3.8 | 0.436 |  | 217 | 1.09 (0.90–1.33) | 0.373 | 0.0 | 3.0 | 0.561 |  | 381 | 0.99 (0.86–1.14) | 0.890 | 32.4 | 5.9 | 0.205 |
|  | Third fourth | 581 | 1.01 (0.89–1.13) | 0.929 | 0.0 | 0.6 | 0.968 |  | 193 | 0.97 (0.79–1.19) | 0.778 | 20.5 | 5.0 | 0.284 |  | 388 | 1.02 (0.89–1.18) | 0.758 | 0.0 | 3.1 | 0.537 |
|  | Highest fourth | 558 | 1.00 (0.88–1.12) | 0.935 | 63.5 | 11.0 | 0.027 |  | 178 | 0.91 (0.74–1.12) | 0.375 | 39.6 | 6.6 | 0.157 |  | 380 | 1.04 (0.90–1.20) | 0.618 | 56.2 | 9.1 | 0.058 |
|  | *Linear trend (per 100mg)* | *2313* | *0.96 (0.90–1.03)* | *0.268* | *57.0* | *9.3* | *0.054* |  | *782* | *0.91 (0.81–1.02)* | *0.094* | *31.8* | *5.9* | *0.209* |  | *1531* | *1.00 (0.92–1.09)* | *0.990* | *35.5* | *6.2* | *0.185* |
|  |  |  |  |  |  |  |  |  |  |  |  |  |  |  |  |  |  |  |  |  |  |
| **DASH score** | Lowest fourth | 562 | 1.00 (reference) | n/a | n/a | n/a | n/a |  | 175 | 1.00 (reference) | n/a | n/a | n/a | n/a |  | 387 | 1.00 (reference) | n/a | n/a | n/a | n/a |
|  | Second fourth | 591 | 1.05 (0.93–1.18) | 0.436 | 0.0 | 1.0 | 0.912 |  | 198 | 1.13 (0.92–1.39) | 0.255 | 0.0 | 2.1 | 0.717 |  | 393 | 1.01 (0.88–1.17) | 0.862 | 0.0 | 2.5 | 0.647 |
|  | Third fourth | 585 | 1.17 (1.04–1.32) | 0.011 | 45.5 | 7.3 | 0.119 |  | 199 | 1.27 (1.03–1.56) | 0.027 | 0.0 | 2.4 | 0.670 |  | 386 | 1.12 (0.97–1.30) | 0.123 | 52.3 | 8.4 | 0.078 |
|  | Highest fourth | 575 | 1.19 (1.05–1.34) | 0.006 | 0.0 | 0.3 | 0.990 |  | 210 | 1.38 (1.12–1.71) | 0.003 | 0.0 | 3.4 | 0.490 |  | 365 | 1.10 (0.95–1.28) | 0.197 | 44.5 | 7.2 | 0.125 |
|  | *Linear trend (per point)* | *2313* | *1.01 (1.01–1.02)* | *0.001* | *0.0* | *1.6* | *0.800* |  | *782* | *1.02 (1.01–1.04)* | *0.007* | *39.9* | *6.7* | *0.155* |  | *1531* | *1.01 (1.00–1.02)* | *0.046* | *55.2* | *8.9* | *0.063* |
|  |  |  |  |  |  |  |  |  |  |  |  |  |  |  |  |  |  |  |  |  |  |
| **aMED score** | 0–2 points | 418 | 1.00 (reference) | n/a | n/a | n/a | n/a |  | 138 | 1.00 (reference) | n/a | n/a | n/a | n/a |  | 280 | 1.00 (reference) | n/a | n/a | n/a | n/a |
|  | 3–4 points | 912 | 1.12 (0.99–1.26) | 0.066 | 64.4 | 11.2 | 0.024 |  | 312 | 1.14 (0.93–1.39) | 0.220 | 0.0 | 3.8 | 0.433 |  | 600 | 1.10 (0.96–1.28) | 0.176 | 57.2 | 9.3 | 0.053 |
|  | 5–6 points | 743 | 1.12 (0.99–1.27) | 0.072 | 18.5 | 4.9 | 0.297 |  | 242 | 1.05 (0.84–1.31) | 0.651 | 46.0 | 7.4 | 0.116 |  | 501 | 1.15 (0.99–1.34) | 0.070 | 61.8 | 10.5 | 0.033 |
|  | 7–9 points | 240 | 1.24 (1.05–1.46) | 0.012 | 42.7 | 7.0 | 0.137 |  | 90 | 1.37 (1.03–1.81) | 0.028 | 0.0 | 2.7 | 0.616 |  | 150 | 1.17 (0.95–1.45) | 0.132 | 51.1 | 8.2 | 0.085 |
|  | *Linear trend (per point)* | *2313* | *1.04 (1.01–1.06)* | *0.004* | *24.1* | *5.3* | *0.261* |  | *782* | *1.05 (1.00–1.09)* | *0.028* | *19.0* | *4.9* | *0.293* |  | *1531* | *1.03 (1.00–1.06)* | *0.045* | *57.3* | *9.4* | *0.053* |
|  |  |  |  |  |  |  |  |  |  |  |  |  |  |  |  |  |  |  |  |  |  |
| **AHEI** | Lowest fourth | 536 | 1.00 (reference) | n/a | n/a | n/a | n/a |  | 172 | 1.00 (reference) | n/a | n/a | n/a | n/a |  | 364 | 1.00 (reference) | n/a | n/a | n/a | n/a |
|  | Second fourth | 578 | 1.06 (0.94–1.19) | 0.325 | 0.0 | 0.3 | 0.990 |  | 178 | 1.01 (0.82–1.25) | 0.928 | 0.0 | 3.7 | 0.447 |  | 400 | 1.08 (0.94–1.25) | 0.270 | 0.0 | 3.9 | 0.415 |
|  | Third fourth | 574 | 1.05 (0.93–1.18) | 0.448 | 0.0 | 3.8 | 0.431 |  | 198 | 1.11 (0.90–1.37) | 0.313 | 0.0 | 2.2 | 0.700 |  | 376 | 1.01 (0.88–1.18) | 0.849 | 26.6 | 5.4 | 0.244 |
|  | Highest fourth | 625 | 1.14 (1.01–1.28) | 0.037 | 0.0 | 3.3 | 0.501 |  | 234 | 1.31 (1.07–1.61) | 0.010 | 53.5 | 8.6 | 0.072 |  | 391 | 1.06 (0.91–1.23) | 0.478 | 35.1 | 6.2 | 0.187 |
|  | *Linear trend (per point)* | *2313* | *1.01 (1.00–1.01)* | *0.004* | *39.4* | *6.6* | *0.158* |  | *782* | *1.01 (1.00–1.02)* | *0.003* | *57.9* | *9.5* | *0.050* |  | *1531* | *1.00 (1.00–1.01)* | *0.157* | *41.0* | *6.8* | *0.148* |
|  |  |  |  |  |  |  |  |  |  |  |  |  |  |  |  |  |  |  |  |  |  |

**Supplementary Table 3** Risk of glioma in relation to intakes of food groups and nutrients and dietary patterns in the Million Women Study, the NIH-AARP Study, and the PLCO Study, stratified by 3 years of follow-up period

|  |  | **First 3 years of follow-up** | | | | | |  | **3+ years of follow-up** | | | | | |
| --- | --- | --- | --- | --- | --- | --- | --- | --- | --- | --- | --- | --- | --- | --- |
| **Dietary factor** | **Intake category** | **No. of cases** | **Summary RR (95% CI)** | **P value** | **Heterogeneity** | | |  | **No. of cases** | **Summary RR (95% CI)** | **P value** | **Heterogeneity** | | |
|  |  |  |  |  | **I^2^ (%)** | **Χ^2^** | **P value** |  |  |  |  | **I^2^ (%)** | **Χ^2^** | **P value** |
| **Total fruit** | Lowest fourth | 99 | 1.00 (reference) | n/a | n/a | n/a | n/a |  | 435 | 1.00 (reference) | n/a | n/a | n/a | n/a |
|  | Second fourth | 104 | 1.02 (0.77–1.35) | 0.912 | 0.0 | 3.0 | 0.556 |  | 423 | 0.94 (0.82–1.08) | 0.411 | 0.0 | 3.0 | 0.554 |
|  | Third fourth | 122 | 1.17 (0.89–1.53) | 0.270 | 0.0 | 0.8 | 0.936 |  | 450 | 1.00 (0.87–1.15) | 0.984 | 0.0 | 3.0 | 0.566 |
|  | Highest fourth | 120 | 1.15 (0.87–1.53) | 0.327 | 25.0 | 5.3 | 0.255 |  | 515 | 1.17 (1.02–1.34) | 0.021 | 0.0 | 2.6 | 0.630 |
|  | *Linear trend (per 100g)* | *445* | *1.06 (1.00–1.12)* | *0.037* | *23.3* | *5.2* | *0.266* |  | *1823* | *1.02 (0.99–1.05)* | *0.220* | *13.9* | *4.6* | *0.326* |
|  |  |  |  |  |  |  |  |  |  |  |  |  |  |  |
| **Citrus fruit** | Lowest fourth | 108 | 1.00 (reference) | n/a | n/a | n/a | n/a |  | 497 | 1.00 (reference) | n/a | n/a | n/a | n/a |
|  | Second fourth | 88 | 1.03 (0.77–1.37) | 0.854 | 0.0 | 3.2 | 0.525 |  | 330 | 0.84 (0.73–0.97) | 0.018 | 0.0 | 2.6 | 0.628 |
|  | Third fourth | 112 | 1.14 (0.87–1.49) | 0.350 | 0.0 | 2.6 | 0.619 |  | 472 | 1.04 (0.92–1.19) | 0.520 | 23.1 | 5.2 | 0.267 |
|  | Highest fourth | 131 | 1.29 (0.98–1.68) | 0.066 | 51.1 | 8.2 | 0.085 |  | 486 | 1.09 (0.96–1.24) | 0.187 | 0.0 | 1.1 | 0.896 |
|  | *Linear trend (per 50g)* | *439* | *1.09 (1.02–1.17)* | *0.016* | *0.0* | *3.5* | *0.477* |  | *1785* | *1.03 (0.98–1.07)* | *0.244* | *30.9* | *5.8* | *0.215* |
|  |  |  |  |  |  |  |  |  |  |  |  |  |  |  |
| **Fruit juice** | Lowest fourth | 110 | 1.00 (reference) | n/a | n/a | n/a | n/a |  | 404 | 1.00 (reference) | n/a | n/a | n/a | n/a |
|  | Second fourth | 95 | 0.86 (0.65–1.14) | 0.287 | 0.0 | 2.2 | 0.702 |  | 455 | 1.10 (0.96–1.26) | 0.157 | 0.0 | 0.4 | 0.982 |
|  | Third fourth | 109 | 0.94 (0.71–1.23) | 0.634 | 51.8 | 8.3 | 0.081 |  | 410 | 0.98 (0.85–1.12) | 0.734 | 12.2 | 4.6 | 0.336 |
|  | Highest fourth | 111 | 1.00 (0.76–1.31) | 0.974 | 14.5 | 4.7 | 0.322 |  | 415 | 0.98 (0.85–1.12) | 0.727 | 0.0 | 1.9 | 0.748 |
|  | *Linear trend (per 100g)* | *425* | *0.99 (0.92–1.06)* | *0.707* | *0.0* | *2.6* | *0.627* |  | *1684* | *1.00 (0.97–1.04)* | *0.873* | *0.0* | *2.7* | *0.612* |
|  |  |  |  |  |  |  |  |  |  |  |  |  |  |  |
| **Total vegetables** | Lowest fourth | 98 | 1.00 (reference) | n/a | n/a | n/a | n/a |  | 406 | 1.00 (reference) | n/a | n/a | n/a | n/a |
|  | Second fourth | 117 | 1.21 (0.92–1.60) | 0.177 | 58.7 | 9.7 | 0.046 |  | 463 | 1.10 (0.97–1.26) | 0.146 | 0.0 | 1.5 | 0.835 |
|  | Third fourth | 128 | 1.27 (0.97–1.66) | 0.084 | 0.0 | 2.6 | 0.621 |  | 470 | 1.12 (0.98–1.28) | 0.101 | 0.0 | 1.2 | 0.881 |
|  | Highest fourth | 108 | 1.08 (0.81–1.43) | 0.614 | 27.4 | 5.5 | 0.239 |  | 487 | 1.18 (1.03–1.35) | 0.019 | 0.0 | 0.9 | 0.925 |
|  | *Linear trend (per 100g)* | *451* | *1.04 (0.97–1.11)* | *0.302* | *0.0* | *1.8* | *0.767* |  | *1826* | *1.03 (0.99–1.07)* | *0.176* | *0.0* | *2.6* | *0.621* |
|  |  |  |  |  |  |  |  |  |  |  |  |  |  |  |
| **Nuts** | Lowest fourth | 139 | 1.00 (reference) | n/a | n/a | n/a | n/a |  | 562 | 1.00 (reference) | n/a | n/a | n/a | n/a |
|  | Second fourth | 97 | 1.07 (0.82–1.41) | 0.605 | 38.5 | 6.5 | 0.165 |  | 371 | 1.16 (1.01–1.34) | 0.031 | 36.5 | 6.3 | 0.178 |
|  | Third fourth | 76 | 0.87 (0.64–1.19) | 0.388 | 62.4 | 10.6 | 0.031 |  | 311 | 1.02 (0.88–1.19) | 0.762 | 32.5 | 5.9 | 0.205 |
|  | Highest fourth | 87 | 1.02 (0.77–1.37) | 0.873 | 39.2 | 6.6 | 0.160 |  | 316 | 1.09 (0.94–1.27) | 0.252 | 43.4 | 7.1 | 0.132 |
|  | *Linear trend (per 10g)* | *399* | *1.00 (0.89–1.12)* | *0.987* | *0.0* | *1.7* | *0.800* |  | *1560* | *1.04 (0.98–1.11)* | *0.172* | *19.3* | *5.0* | *0.292* |
|  |  |  |  |  |  |  |  |  |  |  |  |  |  |  |
| **Grains/ cereal** | Lowest fourth | 109 | 1.00 (reference) | n/a | n/a | n/a | n/a |  | 434 | 1.00 (reference) | n/a | n/a | n/a | n/a |
|  | Second fourth | 109 | 0.96 (0.73–1.26) | 0.756 | 40.7 | 6.7 | 0.150 |  | 457 | 1.02 (0.89–1.17) | 0.749 | 0.0 | 3.3 | 0.509 |
|  | Third fourth | 107 | 0.92 (0.70–1.21) | 0.540 | 0.0 | 2.0 | 0.728 |  | 465 | 1.02 (0.89–1.17) | 0.736 | 44.8 | 7.2 | 0.123 |
|  | Highest fourth | 129 | 1.08 (0.82–1.41) | 0.588 | 46.7 | 7.5 | 0.111 |  | 488 | 1.08 (0.95–1.24) | 0.250 | 0.0 | 2.9 | 0.575 |
|  | *Linear trend (per 50g)* | *454* | *1.08 (0.99–1.18)* | *0.089* | *0.0* | *1.6* | *0.800* |  | *1844* | *1.02 (0.96–1.08)* | *0.586* | *0.0* | *0.2* | *0.996* |
|  |  |  |  |  |  |  |  |  |  |  |  |  |  |  |
| **Red meat** | Lowest fourth | 114 | 1.00 (reference) | n/a | n/a | n/a | n/a |  | 459 | 1.00 (reference) | n/a | n/a | n/a | n/a |
|  | Second fourth | 130 | 1.12 (0.87–1.45) | 0.380 | 0.0 | 2.3 | 0.682 |  | 477 | 1.03 (0.90–1.17) | 0.685 | 49.9 | 8.0 | 0.092 |
|  | Third fourth | 118 | 1.03 (0.79–1.34) | 0.842 | 0.0 | 0.7 | 0.953 |  | 415 | 0.90 (0.79–1.03) | 0.135 | 38.2 | 6.5 | 0.166 |
|  | Highest fourth | 81 | 0.73 (0.54–0.97) | 0.031 | 0.0 | 1.6 | 0.817 |  | 449 | 0.99 (0.86–1.13) | 0.851 | 65.4 | 11.6 | 0.021 |
|  | *Linear trend (per 50g)* | *443* | *0.80 (0.67–0.95)* | *0.010* | *0.0* | *2.4* | *0.670* |  | *1800* | *0.99 (0.91–1.08)* | *0.880* | *59.1* | *9.8* | *0.045* |
|  |  |  |  |  |  |  |  |  |  |  |  |  |  |  |
| **Processed meat** | Lowest fourth | 134 | 1.00 (reference) | n/a | n/a | n/a | n/a |  | 523 | 1.00 (reference) | n/a | n/a | n/a | n/a |
|  | Second fourth | 104 | 1.00 (0.77–1.29) | 0.973 | 0.0 | 1.4 | 0.843 |  | 396 | 1.04 (0.91–1.19) | 0.593 | 0.0 | 1.9 | 0.758 |
|  | Third fourth | 112 | 0.93 (0.72–1.20) | 0.587 | 0.0 | 1.3 | 0.859 |  | 470 | 1.03 (0.91–1.17) | 0.619 | 0.0 | 2.4 | 0.665 |
|  | Highest fourth | 93 | 0.78 (0.60–1.03) | 0.078 | 0.0 | 2.6 | 0.621 |  | 411 | 0.92 (0.81–1.05) | 0.209 | 15.2 | 4.7 | 0.318 |
|  | *Linear trend (per 10g)* | *443* | *0.94 (0.87–1.01)* | *0.078* | *0.0* | *2.9* | *0.583* |  | *1800* | *0.99 (0.96–1.03)* | *0.769* | *6.9* | *4.3* | *0.367* |
|  |  |  |  |  |  |  |  |  |  |  |  |  |  |  |
| **White meat** | Lowest fourth | 119 | 1.00 (reference) | n/a | n/a | n/a | n/a |  | 433 | 1.00 (reference) | n/a | n/a | n/a | n/a |
|  | Second fourth | 119 | 1.00 (0.77–1.29) | 0.994 | 0.0 | 2.7 | 0.602 |  | 475 | 1.08 (0.94–1.23) | 0.278 | 0.0 | 3.5 | 0.475 |
|  | Third fourth | 108 | 0.93 (0.72–1.21) | 0.601 | 0.0 | 0.5 | 0.975 |  | 456 | 1.03 (0.90–1.17) | 0.704 | 47.6 | 7.6 | 0.106 |
|  | Highest fourth | 97 | 0.87 (0.66–1.14) | 0.308 | 0.0 | 2.5 | 0.642 |  | 436 | 0.99 (0.86–1.13) | 0.853 | 0.0 | 2.3 | 0.689 |
|  | *Linear trend (per 50g)* | *443* | *0.96 (0.83–1.11)* | *0.592* | *0.0* | *3.3* | *0.507* |  | *1800* | *0.98 (0.91–1.05)* | *0.525* | *0.0* | *1.1* | *0.889* |
|  |  |  |  |  |  |  |  |  |  |  |  |  |  |  |
| **Fish** | Lowest fourth | 113 | 1.00 (reference) | n/a | n/a | n/a | n/a |  | 429 | 1.00 (reference) | n/a | n/a | n/a | n/a |
|  | Second fourth | 119 | 1.07 (0.82–1.38) | 0.636 | 5.7 | 4.2 | 0.374 |  | 470 | 1.07 (0.94–1.22) | 0.314 | 55.2 | 8.9 | 0.063 |
|  | Third fourth | 106 | 0.96 (0.73–1.25) | 0.737 | 0.0 | 0.9 | 0.930 |  | 467 | 1.04 (0.91–1.19) | 0.545 | 70.3 | 13.4 | 0.009 |
|  | Highest fourth | 109 | 0.99 (0.75–1.30) | 0.937 | 0.0 | 2.6 | 0.625 |  | 433 | 0.97 (0.85–1.12) | 0.714 | 52.0 | 8.3 | 0.080 |
|  | *Linear trend (per 50g)* | *447* | *0.95 (0.69–1.29)* | *0.730* | *0.0* | *1.7* | *0.795* |  | *1799* | *0.99 (0.84–1.16)* | *0.882* | *19.5* | *5.0* | *0.290* |
|  |  |  |  |  |  |  |  |  |  |  |  |  |  |  |
| **Eggs** | Lowest fourth | 109 | 1.00 (reference) | n/a | n/a | n/a | n/a |  | 458 | 1.00 (reference) | n/a | n/a | n/a | n/a |
|  | Second fourth | 115 | 1.04 (0.80–1.36) | 0.770 | 0.0 | 2.4 | 0.669 |  | 420 | 0.91 (0.79–1.04) | 0.155 | 0.0 | 0.8 | 0.942 |
|  | Third fourth | 115 | 1.06 (0.81–1.38) | 0.682 | 21.0 | 5.1 | 0.281 |  | 498 | 1.07 (0.94–1.22) | 0.294 | 0.0 | 0.7 | 0.952 |
|  | Highest fourth | 112 | 1.04 (0.79–1.36) | 0.789 | 0.0 | 3.8 | 0.434 |  | 463 | 1.04 (0.91–1.18) | 0.606 | 0.0 | 2.0 | 0.740 |
|  | *Linear trend (per 10g)* | *451* | *0.99 (0.92–1.08)* | *0.889* | *46.0* | *7.4* | *0.116* |  | *1839* | *1.00 (0.95–1.05)* | *0.942* | *0.1* | *4.0* | *0.405* |
|  |  |  |  |  |  |  |  |  |  |  |  |  |  |  |
| **Dairy products** | Lowest fourth | 115 | 1.00 (reference) | n/a | n/a | n/a | n/a |  | 437 | 1.00 (reference) | n/a | n/a | n/a | n/a |
|  | Second fourth | 126 | 1.03 (0.79–1.33) | 0.844 | 47.0 | 7.5 | 0.110 |  | 461 | 1.01 (0.88–1.15) | 0.926 | 19.0 | 4.9 | 0.294 |
|  | Third fourth | 119 | 0.97 (0.75–1.26) | 0.826 | 36.4 | 6.3 | 0.178 |  | 483 | 1.05 (0.92–1.20) | 0.463 | 0.0 | 0.4 | 0.980 |
|  | Highest fourth | 97 | 0.78 (0.59–1.04) | 0.087 | 31.7 | 5.9 | 0.210 |  | 475 | 1.04 (0.91–1.18) | 0.611 | 0.0 | 2.0 | 0.743 |
|  | *Linear trend (per 100g)* | *457* | *1.02 (0.98–1.06)* | *0.457* | *17.7* | *4.9* | *0.302* |  | *1856* | *1.00 (0.97–1.02)* | *0.875* | *53.5* | *8.6* | *0.072* |
|  |  |  |  |  |  |  |  |  |  |  |  |  |  |  |
| **Cheese** | Lowest fourth | 111 | 1.00 (reference) | n/a | n/a | n/a | n/a |  | 435 | 1.00 (reference) | n/a | n/a | n/a | n/a |
|  | Second fourth | 101 | 0.88 (0.67–1.16) | 0.361 | 0.0 | 2.3 | 0.679 |  | 435 | 0.99 (0.86–1.13) | 0.866 | 0.0 | 1.8 | 0.781 |
|  | Third fourth | 102 | 0.90 (0.69–1.19) | 0.471 | 0.0 | 0.9 | 0.921 |  | 432 | 0.98 (0.85–1.12) | 0.751 | 0.0 | 2.3 | 0.687 |
|  | Highest fourth | 125 | 1.10 (0.85–1.43) | 0.474 | 22.2 | 5.1 | 0.273 |  | 466 | 1.06 (0.93–1.21) | 0.393 | 0.0 | 3.0 | 0.564 |
|  | *Linear trend (per 10g)* | *439* | *1.06 (0.92–1.22)* | *0.413* | *18.6* | *4.9* | *0.296* |  | *1768* | *1.03 (0.95–1.11)* | *0.486* | *8.5* | *4.4* | *0.358* |
|  |  |  |  |  |  |  |  |  |  |  |  |  |  |  |
| **Coffee** | Lowest fourth | 110 | 1.00 (reference) | n/a | n/a | n/a | n/a |  | 419 | 1.00 (reference) | n/a | n/a | n/a | n/a |
|  | Second fourth | 111 | 1.06 (0.81–1.39) | 0.665 | 0.0 | 0.4 | 0.986 |  | 428 | 1.00 (0.87–1.15) | 0.951 | 0.0 | 2.0 | 0.732 |
|  | Third fourth | 110 | 1.06 (0.80–1.40) | 0.699 | 0.0 | 1.7 | 0.797 |  | 439 | 1.03 (0.89–1.18) | 0.731 | 41.6 | 6.9 | 0.144 |
|  | Highest fourth | 97 | 0.96 (0.71–1.28) | 0.759 | 0.0 | 1.5 | 0.824 |  | 420 | 1.03 (0.89–1.19) | 0.680 | 0.8 | 4.0 | 0.401 |
|  | *Linear trend (per 100g)* | *428* | *0.99 (0.97–1.00)* | *0.133* | *0.0* | *1.2* | *0.881* |  | *1706* | *1.01 (1.00–1.02)* | *0.128* | *0.0* | *3.7* | *0.453* |
|  |  |  |  |  |  |  |  |  |  |  |  |  |  |  |
| **Tea** | Lowest fourth | 108 | 1.00 (reference) | n/a | n/a | n/a | n/a |  | 434 | 1.00 (reference) | n/a | n/a | n/a | n/a |
|  | Second fourth | 124 | 1.17 (0.90–1.52) | 0.230 | 0.0 | 0.6 | 0.958 |  | 433 | 0.96 (0.84–1.10) | 0.541 | 26.9 | 5.5 | 0.242 |
|  | Third fourth | 109 | 1.03 (0.78–1.35) | 0.847 | 0.0 | 2.1 | 0.722 |  | 436 | 0.96 (0.84–1.09) | 0.514 | 0.0 | 1.7 | 0.793 |
|  | Highest fourth | 87 | 0.84 (0.62–1.12) | 0.230 | 0.0 | 2.5 | 0.646 |  | 476 | 1.05 (0.91–1.20) | 0.519 | 0.0 | 1.6 | 0.809 |
|  | *Linear trend (per 100g)* | *428* | *1.00 (0.98–1.03)* | *0.929* | *0.0* | *2.7* | *0.602* |  | *1779* | *1.00 (0.99–1.02)* | *0.778* | *0.0* | *2.2* | *0.693* |
|  |  |  |  |  |  |  |  |  |  |  |  |  |  |  |
| **Carbo-hydrate** | Lowest fourth | 102 | 1.00 (reference) | n/a | n/a | n/a | n/a |  | 413 | 1.00 (reference) | n/a | n/a | n/a | n/a |
|  | Second fourth | 96 | 0.94 (0.70–1.25) | 0.659 | 0.0 | 2.6 | 0.626 |  | 485 | 1.14 (0.99–1.30) | 0.062 | 0.0 | 3.0 | 0.556 |
|  | Third fourth | 131 | 1.28 (0.97–1.69) | 0.078 | 0.0 | 2.4 | 0.659 |  | 473 | 1.09 (0.95–1.26) | 0.203 | 46.9 | 7.5 | 0.110 |
|  | Highest fourth | 128 | 1.27 (0.95–1.69) | 0.110 | 37.1 | 6.4 | 0.174 |  | 485 | 1.14 (0.99–1.32) | 0.070 | 0.0 | 2.9 | 0.583 |
|  | *Linear trend (per 1% energy)* | *457* | *1.02 (1.00–1.03)* | *0.039* | *5.4* | *4.2* | *0.376* |  | *1856* | *1.01 (1.00–1.01)* | *0.187* | *8.3* | *4.4* | *0.359* |
|  |  |  |  |  |  |  |  |  |  |  |  |  |  |  |
| **Protein** | Lowest fourth | 132 | 1.00 (reference) | n/a | n/a | n/a | n/a |  | 456 | 1.00 (reference) | n/a | n/a | n/a | n/a |
|  | Second fourth | 130 | 0.95 (0.74–1.21) | 0.678 | 0.0 | 3.7 | 0.452 |  | 455 | 0.97 (0.85–1.10) | 0.626 | 0.0 | 1.6 | 0.816 |
|  | Third fourth | 111 | 0.81 (0.63–1.05) | 0.118 | 0.0 | 3.8 | 0.434 |  | 487 | 1.03 (0.90–1.17) | 0.649 | 0.0 | 3.0 | 0.552 |
|  | Highest fourth | 84 | 0.62 (0.47–0.83) | 0.001 | 0.0 | 2.3 | 0.690 |  | 458 | 0.98 (0.85–1.12) | 0.725 | 0.0 | 1.5 | 0.832 |
|  | *Linear trend (per 1% energy)* | *457* | *0.95 (0.91–0.99)* | *0.014* | *16.8* | *4.8* | *0.307* |  | *1856* | *1.00 (0.98–1.02)* | *0.890* | *0.0* | *1.7* | *0.788* |
|  |  |  |  |  |  |  |  |  |  |  |  |  |  |  |
| **Total fat** | Lowest fourth | 114 | 1.00 (reference) | n/a | n/a | n/a | n/a |  | 476 | 1.00 (reference) | n/a | n/a | n/a | n/a |
|  | Second fourth | 113 | 0.96 (0.74–1.25) | 0.757 | 0.0 | 0.4 | 0.985 |  | 471 | 0.98 (0.86–1.12) | 0.778 | 0.0 | 0.2 | 0.997 |
|  | Third fourth | 107 | 0.89 (0.68–1.17) | 0.408 | 0.0 | 1.1 | 0.897 |  | 458 | 0.96 (0.84–1.10) | 0.582 | 0.0 | 3.4 | 0.486 |
|  | Highest fourth | 123 | 1.03 (0.78–1.35) | 0.842 | 22.1 | 5.1 | 0.273 |  | 451 | 0.97 (0.85–1.11) | 0.702 | 0.0 | 3.1 | 0.540 |
|  | *Linear trend (per 1% energy)* | *457* | *1.00 (0.98–1.01)* | *0.778* | *24.3* | *5.3* | *0.259* |  | *1856* | *1.00 (0.99–1.01)* | *0.733* | *0.0* | *1.6* | *0.812* |
|  |  |  |  |  |  |  |  |  |  |  |  |  |  |  |
| **Saturated fat** | Lowest fourth | 110 | 1.00 (reference) | n/a | n/a | n/a | n/a |  | 462 | 1.00 (reference) | n/a | n/a | n/a | n/a |
|  | Second fourth | 114 | 1.00 (0.77–1.31) | 0.993 | 25.0 | 5.3 | 0.255 |  | 474 | 1.02 (0.90–1.16) | 0.729 | 0.0 | 1.1 | 0.895 |
|  | Third fourth | 112 | 0.98 (0.74–1.29) | 0.879 | 15.7 | 4.7 | 0.314 |  | 464 | 1.01 (0.88–1.15) | 0.929 | 0.0 | 2.4 | 0.659 |
|  | Highest fourth | 121 | 1.06 (0.81–1.39) | 0.687 | 0.0 | 1.1 | 0.894 |  | 456 | 1.01 (0.88–1.16) | 0.908 | 39.8 | 6.6 | 0.156 |
|  | *Linear trend (per 1% energy)* | *457* | *0.99 (0.95–1.04)* | *0.712* | *0.0* | *3.7* | *0.447* |  | *1856* | *1.00 (0.97–1.02)* | *0.838* | *0.0* | *3.7* | *0.442* |
|  |  |  |  |  |  |  |  |  |  |  |  |  |  |  |
| **Mono-unsaturated fat** | Lowest fourth | 105 | 1.00 (reference) | n/a | n/a | n/a | n/a |  | 490 | 1.00 (reference) | n/a | n/a | n/a | n/a |
|  | Second fourth | 122 | 1.13 (0.87–1.48) | 0.353 | 0.0 | 1.4 | 0.842 |  | 467 | 0.95 (0.83–1.08) | 0.403 | 0.0 | 0.9 | 0.931 |
|  | Third fourth | 105 | 0.97 (0.73–1.27) | 0.807 | 0.0 | 1.3 | 0.853 |  | 472 | 0.96 (0.85–1.10) | 0.572 | 0.0 | 2.1 | 0.715 |
|  | Highest fourth | 125 | 1.14 (0.86–1.50) | 0.356 | 26.8 | 5.5 | 0.243 |  | 427 | 0.90 (0.78–1.03) | 0.115 | 0.0 | 1.5 | 0.831 |
|  | *Linear trend (per 1% energy)* | *457* | *0.99 (0.95–1.03)* | *0.615* | *47.0* | *7.6* | *0.109* |  | *1856* | *0.99 (0.97–1.02)* | *0.625* | *0.0* | *3.7* | *0.444* |
|  |  |  |  |  |  |  |  |  |  |  |  |  |  |  |
| **Poly-unsaturated fat** | Lowest fourth | 110 | 1.00 (reference) | n/a | n/a | n/a | n/a |  | 452 | 1.00 (reference) | n/a | n/a | n/a | n/a |
|  | Second fourth | 106 | 0.94 (0.72–1.24) | 0.677 | 0.0 | 3.0 | 0.561 |  | 509 | 1.11 (0.97–1.26) | 0.118 | 41.6 | 6.9 | 0.144 |
|  | Third fourth | 120 | 1.07 (0.82–1.39) | 0.614 | 0.0 | 2.1 | 0.720 |  | 441 | 0.96 (0.84–1.10) | 0.573 | 0.0 | 4.0 | 0.407 |
|  | Highest fourth | 121 | 1.09 (0.83–1.42) | 0.534 | 44.4 | 7.2 | 0.126 |  | 454 | 1.00 (0.88–1.15) | 0.946 | 0.0 | 1.0 | 0.910 |
|  | *Linear trend (per 1% energy)* | *457* | *1.04 (0.98–1.10)* | *0.197* | *52.7* | *8.4* | *0.076* |  | *1856* | *0.99 (0.96–1.03)* | *0.724* | *0.0* | *1.2* | *0.871* |
|  |  |  |  |  |  |  |  |  |  |  |  |  |  |  |
| **Alcohol** | Lowest fourth | 171 | 1.00 (reference) | n/a | n/a | n/a | n/a |  | 620 | 1.00 (reference) | n/a | n/a | n/a | n/a |
|  | Second fourth | 95 | 0.91 (0.69–1.19) | 0.477 | 23.0 | 5.2 | 0.268 |  | 361 | 1.03 (0.90–1.18) | 0.660 | 32.3 | 5.9 | 0.206 |
|  | Third fourth | 97 | 0.83 (0.64–1.07) | 0.152 | 0.0 | 4.0 | 0.408 |  | 457 | 1.12 (0.98–1.27) | 0.088 | 0.0 | 0.9 | 0.922 |
|  | Highest fourth | 94 | 0.81 (0.62–1.06) | 0.130 | 0.0 | 1.1 | 0.887 |  | 418 | 1.08 (0.95–1.23) | 0.240 | 0.0 | 2.6 | 0.635 |
|  | *Linear trend (per 10g)* | *457* | *0.96 (0.90–1.03)* | *0.249* | *0.0* | *2.5* | *0.636* |  | *1856* | *0.98 (0.94–1.02)* | *0.265* | *30.3* | *5.7* | *0.220* |
|  |  |  |  |  |  |  |  |  |  |  |  |  |  |  |
| **Fibre** | Lowest fourth | 92 | 1.00 (reference) | n/a | n/a | n/a | n/a |  | 421 | 1.00 (reference) | n/a | n/a | n/a | n/a |
|  | Second fourth | 119 | 1.25 (0.94–1.65) | 0.119 | 0.0 | 2.6 | 0.622 |  | 447 | 1.02 (0.89–1.17) | 0.750 | 0.0 | 3.6 | 0.465 |
|  | Third fourth | 118 | 1.22 (0.92–1.61) | 0.173 | 0.0 | 1.8 | 0.777 |  | 487 | 1.11 (0.97–1.26) | 0.146 | 47.2 | 7.6 | 0.108 |
|  | Highest fourth | 128 | 1.31 (0.98–1.74) | 0.064 | 0.0 | 3.7 | 0.454 |  | 501 | 1.14 (1.00–1.31) | 0.054 | 0.0 | 3.8 | 0.429 |
|  | *Linear trend (per 5g)* | *457* | *1.14 (1.01–1.28)* | *0.036* | *0.0* | *1.1* | *0.897* |  | *1856* | *1.07 (1.00–1.14)* | *0.054* | *0.0* | *3.5* | *0.481* |
|  |  |  |  |  |  |  |  |  |  |  |  |  |  |  |
| **Carotene** | Lowest fourth | 94 | 1.00 (reference) | n/a | n/a | n/a | n/a |  | 429 | 1.00 (reference) | n/a | n/a | n/a | n/a |
|  | Second fourth | 119 | 1.24 (0.94–1.63) | 0.133 | 5.9 | 4.3 | 0.373 |  | 504 | 1.15 (1.01–1.31) | 0.041 | 29.5 | 5.7 | 0.225 |
|  | Third fourth | 108 | 1.12 (0.84–1.49) | 0.453 | 32.4 | 5.9 | 0.206 |  | 441 | 1.00 (0.88–1.15) | 0.951 | 0.0 | 3.2 | 0.521 |
|  | Highest fourth | 136 | 1.40 (1.06–1.84) | 0.017 | 11.4 | 4.5 | 0.341 |  | 482 | 1.11 (0.97–1.27) | 0.122 | 0.0 | 1.0 | 0.917 |
|  | *Linear trend (per 1000ug)* | *457* | *1.07 (0.94–1.22)* | *0.327* | *0.0* | *1.5* | *0.822* |  | *1856* | *1.02 (0.95–1.09)* | *0.639* | *0.0* | *2.5* | *0.644* |
|  |  |  |  |  |  |  |  |  |  |  |  |  |  |  |
| **Vitamin C** | Lowest fourth | 104 | 1.00 (reference) | n/a | n/a | n/a | n/a |  | 445 | 1.00 (reference) | n/a | n/a | n/a | n/a |
|  | Second fourth | 114 | 1.07 (0.82–1.40) | 0.629 | 0.0 | 3.1 | 0.539 |  | 427 | 0.92 (0.81–1.06) | 0.249 | 21.1 | 5.1 | 0.280 |
|  | Third fourth | 117 | 1.08 (0.82–1.42) | 0.604 | 44.5 | 7.2 | 0.126 |  | 509 | 1.09 (0.96–1.25) | 0.177 | 0.0 | 2.0 | 0.729 |
|  | Highest fourth | 122 | 1.14 (0.87–1.50) | 0.341 | 0.0 | 3.8 | 0.435 |  | 475 | 1.04 (0.91–1.19) | 0.564 | 0.0 | 2.8 | 0.586 |
|  | *Linear trend (per 50mg)* | *457* | *1.03 (0.97–1.09)* | *0.362* | *0.0* | *3.6* | *0.460* |  | *1856* | *1.01 (0.98–1.05)* | *0.395* | *0.0* | *3.1* | *0.538* |
|  |  |  |  |  |  |  |  |  |  |  |  |  |  |  |
| **Vitamin E** | Lowest fourth | 100 | 1.00 (reference) | n/a | n/a | n/a | n/a |  | 455 | 1.00 (reference) | n/a | n/a | n/a | n/a |
|  | Second fourth | 113 | 1.11 (0.84–1.45) | 0.476 | 34.7 | 6.1 | 0.190 |  | 466 | 0.99 (0.87–1.13) | 0.915 | 28.4 | 5.6 | 0.232 |
|  | Third fourth | 124 | 1.22 (0.93–1.59) | 0.153 | 5.2 | 4.2 | 0.377 |  | 470 | 1.00 (0.88–1.14) | 0.990 | 0.0 | 2.6 | 0.621 |
|  | Highest fourth | 120 | 1.19 (0.90–1.56) | 0.219 | 28.9 | 5.6 | 0.229 |  | 465 | 1.00 (0.88–1.14) | 0.976 | 0.0 | 3.9 | 0.420 |
|  | *Linear trend (per 5mg)* | *457* | *1.02 (0.87–1.20)* | *0.808* | *0.0* | *1.5* | *0.824* |  | *1856* | *1.01 (0.92–1.10)* | *0.882* | *0.0* | *2.6* | *0.629* |
|  |  |  |  |  |  |  |  |  |  |  |  |  |  |  |
| **Folate** | Lowest fourth | 120 | 1.00 (reference) | n/a | n/a | n/a | n/a |  | 409 | 1.00 (reference) | n/a | n/a | n/a | n/a |
|  | Second fourth | 116 | 0.93 (0.72–1.21) | 0.604 | 0.0 | 0.9 | 0.928 |  | 450 | 1.06 (0.93–1.22) | 0.370 | 0.0 | 0.8 | 0.940 |
|  | Third fourth | 107 | 0.85 (0.65–1.11) | 0.227 | 0.0 | 0.9 | 0.926 |  | 490 | 1.15 (1.00–1.31) | 0.044 | 0.0 | 0.3 | 0.989 |
|  | Highest fourth | 114 | 0.90 (0.68–1.18) | 0.446 | 27.9 | 5.5 | 0.236 |  | 507 | 1.19 (1.04–1.37) | 0.011 | 0.0 | 2.1 | 0.712 |
|  | *Linear trend (per 50ug)* | *457* | *1.02 (0.98–1.06)* | *0.318* | *50.4* | *8.1* | *0.089* |  | *1856* | *1.01 (0.99–1.03)* | *0.222* | *54.5* | *8.8* | *0.066* |
|  |  |  |  |  |  |  |  |  |  |  |  |  |  |  |
| **Calcium** | Lowest fourth | 111 | 1.00 (reference) | n/a | n/a | n/a | n/a |  | 454 | 1.00 (reference) | n/a | n/a | n/a | n/a |
|  | Second fourth | 123 | 1.05 (0.81–1.36) | 0.728 | 0.0 | 3.9 | 0.422 |  | 453 | 0.96 (0.84–1.09) | 0.502 | 23.1 | 5.2 | 0.267 |
|  | Third fourth | 122 | 1.02 (0.79–1.33) | 0.859 | 0.0 | 2.5 | 0.653 |  | 454 | 0.95 (0.83–1.09) | 0.465 | 0.0 | 1.4 | 0.853 |
|  | Highest fourth | 101 | 0.83 (0.62–1.09) | 0.185 | 41.8 | 6.9 | 0.143 |  | 495 | 1.04 (0.92–1.19) | 0.526 | 20.7 | 5.0 | 0.283 |
|  | *Linear trend (per 100mg)* | *457* | *1.01 (0.98–1.05)* | *0.504* | *44.9* | *7.3* | *0.123* |  | *1856* | *1.00 (0.98–1.02)* | *0.921* | *59.3* | *9.8* | *0.044* |
|  |  |  |  |  |  |  |  |  |  |  |  |  |  |  |
| **Cholesterol** | Lowest fourth | 113 | 1.00 (reference) | n/a | n/a | n/a | n/a |  | 463 | 1.00 (reference) | n/a | n/a | n/a | n/a |
|  | Second fourth | 127 | 1.10 (0.85–1.43) | 0.446 | 0.0 | 2.2 | 0.705 |  | 471 | 1.01 (0.89–1.15) | 0.909 | 0.0 | 3.6 | 0.459 |
|  | Third fourth | 108 | 0.94 (0.72–1.23) | 0.662 | 10.5 | 4.5 | 0.346 |  | 473 | 1.02 (0.90–1.16) | 0.760 | 0.0 | 1.5 | 0.830 |
|  | Highest fourth | 109 | 0.97 (0.74–1.28) | 0.825 | 49.3 | 7.9 | 0.096 |  | 449 | 1.00 (0.88–1.14) | 0.986 | 55.6 | 9.0 | 0.061 |
|  | *Linear trend (per 100mg)* | *457* | *0.91 (0.79–1.06)* | *0.220* | *47.7* | *7.7* | *0.105* |  | *1856* | *0.98 (0.91–1.06)* | *0.681* | *36.2* | *6.3* | *0.180* |
|  |  |  |  |  |  |  |  |  |  |  |  |  |  |  |
| **DASH score** | Lowest fourth | 106 | 1.00 (reference) | n/a | n/a | n/a | n/a |  | 456 | 1.00 (reference) | n/a | n/a | n/a | n/a |
|  | Second fourth | 121 | 1.14 (0.88–1.49) | 0.326 | 0.0 | 0.5 | 0.971 |  | 470 | 1.03 (0.90–1.17) | 0.709 | 0.0 | 1.2 | 0.870 |
|  | Third fourth | 112 | 1.16 (0.89–1.53) | 0.276 | 0.0 | 1.8 | 0.778 |  | 473 | 1.17 (1.02–1.33) | 0.021 | 42.8 | 7.0 | 0.137 |
|  | Highest fourth | 118 | 1.28 (0.97–1.69) | 0.081 | 0.0 | 3.5 | 0.476 |  | 457 | 1.16 (1.02–1.33) | 0.029 | 0.0 | 2.5 | 0.639 |
|  | *Linear trend (per point)* | *457* | *1.01 (0.99–1.03)* | *0.262* | *54.5* | *8.8* | *0.066* |  | *1856* | *1.01 (1.01–1.02)* | *0.003* | *25.6* | *5.4* | *0.251* |
|  |  |  |  |  |  |  |  |  |  |  |  |  |  |  |
| **aMED score** | 0–2 points | 77 | 1.00 (reference) | n/a | n/a | n/a | n/a |  | 341 | 1.00 (reference) | n/a | n/a | n/a | n/a |
|  | 3–4 points | 196 | 1.26 (0.96–1.65) | 0.097 | 9.5 | 4.4 | 0.352 |  | 716 | 1.08 (0.95–1.23) | 0.237 | 51.3 | 8.2 | 0.084 |
|  | 5–6 points | 130 | 1.00 (0.74–1.35) | 0.991 | 27.2 | 5.5 | 0.240 |  | 613 | 1.15 (1.00–1.32) | 0.050 | 0.0 | 3.5 | 0.473 |
|  | 7–9 points | 54 | 1.45 (1.00–2.09) | 0.050 | 0.0 | 2.8 | 0.593 |  | 186 | 1.19 (0.99–1.44) | 0.068 | 43.1 | 7.0 | 0.134 |
|  | *Linear trend (per point)* | *457* | *1.03 (0.98–1.09)* | *0.282* | *19.7* | *5.0* | *0.289* |  | *1856* | *1.04 (1.01–1.07)* | *0.007* | *34.9* | *6.1* | *0.188* |
|  |  |  |  |  |  |  |  |  |  |  |  |  |  |  |
| **AHEI** | Lowest fourth | 98 | 1.00 (reference) | n/a | n/a | n/a | n/a |  | 438 | 1.00 (reference) | n/a | n/a | n/a | n/a |
|  | Second fourth | 108 | 1.07 (0.81–1.42) | 0.622 | 8.0 | 4.3 | 0.361 |  | 470 | 1.06 (0.93–1.21) | 0.410 | 0.0 | 2.6 | 0.620 |
|  | Third fourth | 121 | 1.19 (0.90–1.57) | 0.225 | 52.3 | 8.4 | 0.078 |  | 453 | 1.01 (0.89–1.16) | 0.859 | 9.7 | 4.4 | 0.351 |
|  | Highest fourth | 130 | 1.26 (0.95–1.67) | 0.109 | 78.2 | 18.4 | 0.001 |  | 495 | 1.10 (0.97–1.26) | 0.149 | 0.0 | 2.9 | 0.574 |
|  | *Linear trend (per point)* | *457* | *1.01 (1.00–1.02)* | *0.052* | *81.3* | *21.4* | *<0.001* |  | *1856* | *1.00 (1.00–1.01)* | *0.025* | *11.9* | *4.5* | *0.338* |

**Supplementary Figure 1** Participants inclusion/exclusion flow diagram in the Million Women Study, the NIH-AARP Study, and the PLCO Study


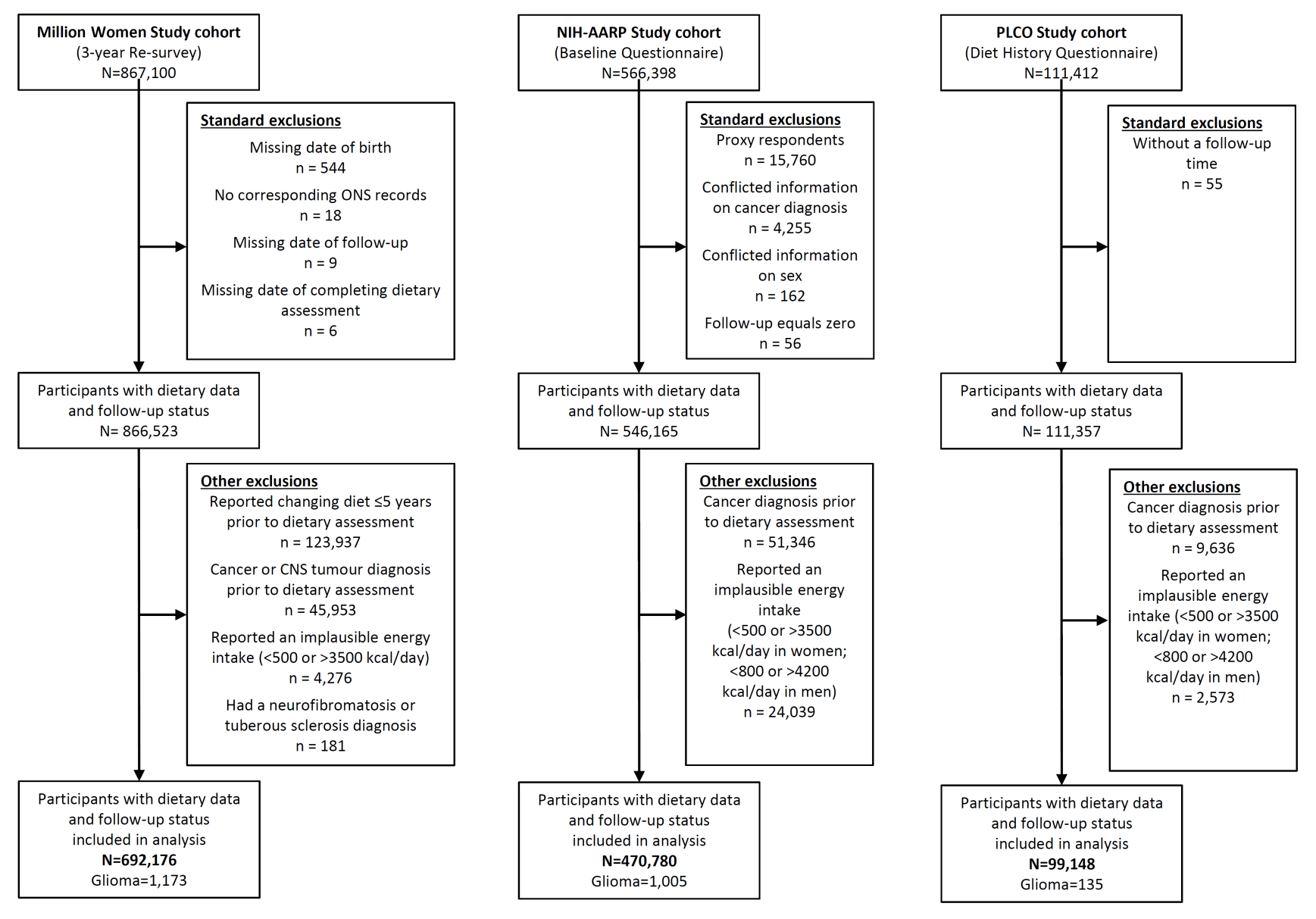


**Supplementary Figure 2a** Risk of glioma in relation to increasing intakes of food groups and nutrients in the Million Women Study


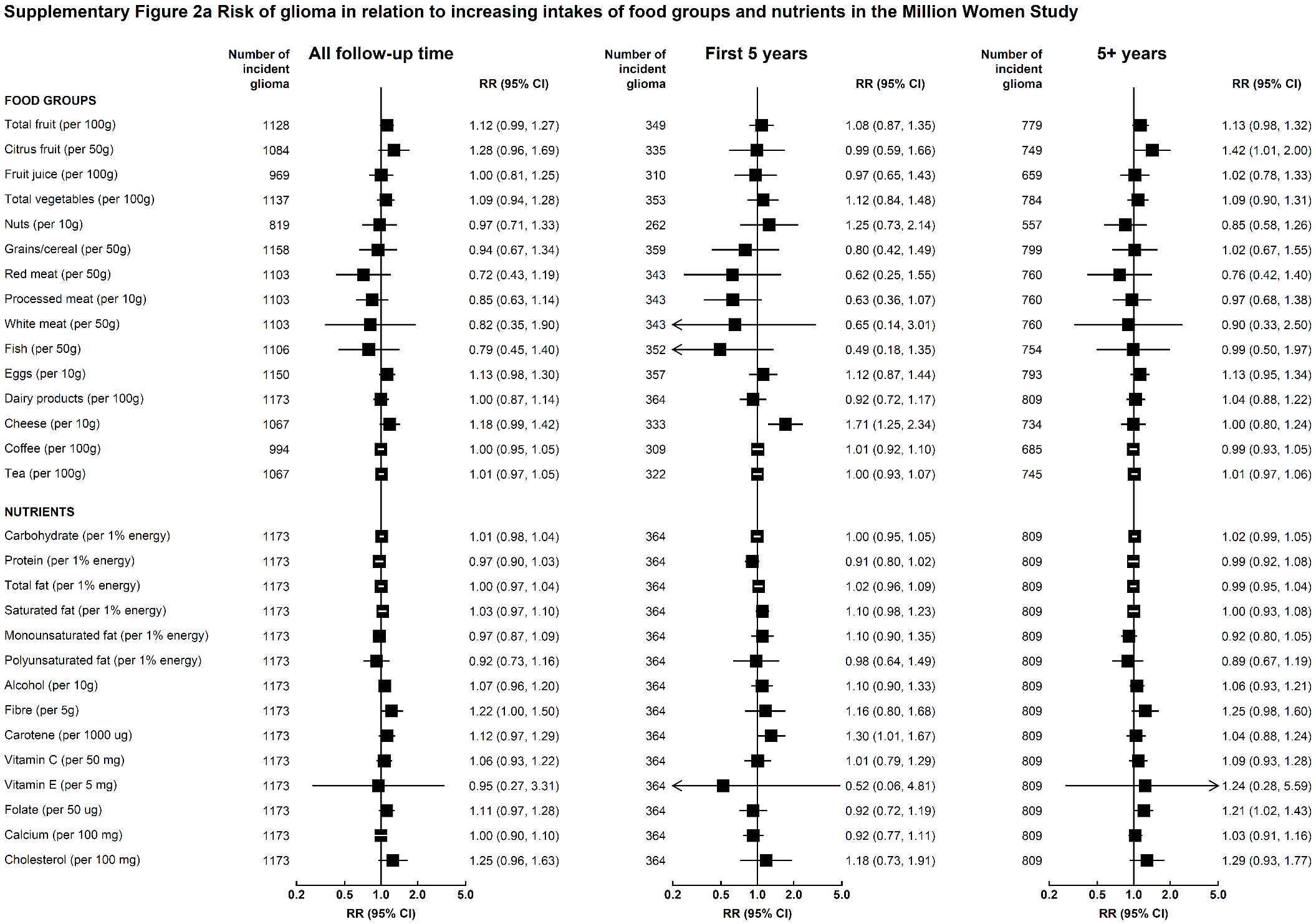


**Supplementary Figure 2b** Risk of glioma in relation to increasing intakes of food groups and nutrients of men in the NIH-AARP Study


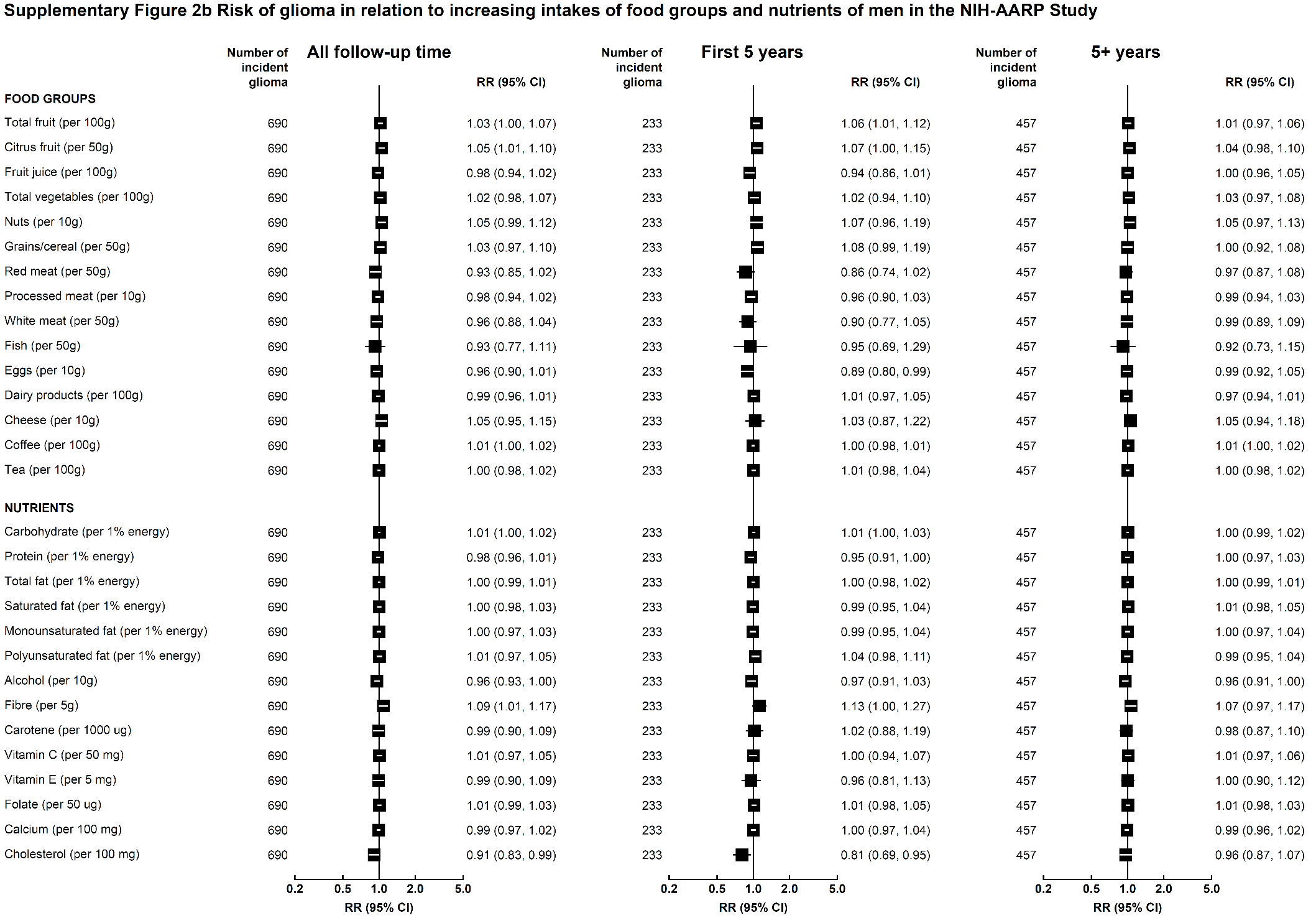


**Supplementary Figure 2c** Risk of glioma in relation to increasing intakes of food groups and nutrients of women in the NIH-AARP Study


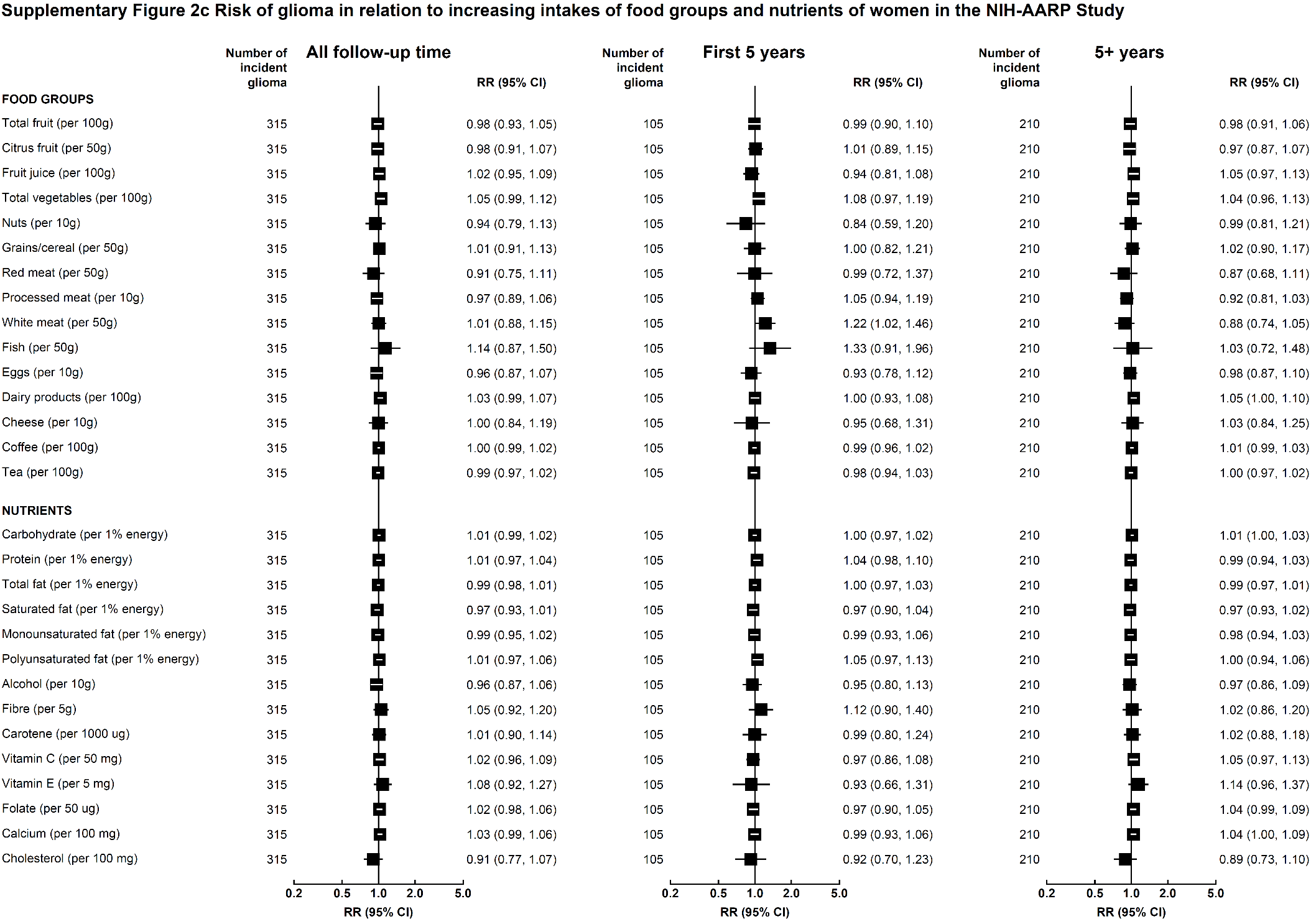


**Supplementary Figure 2d** Risk of glioma in relation to increasing intakes of food groups and nutrients of men in the PLCO Study


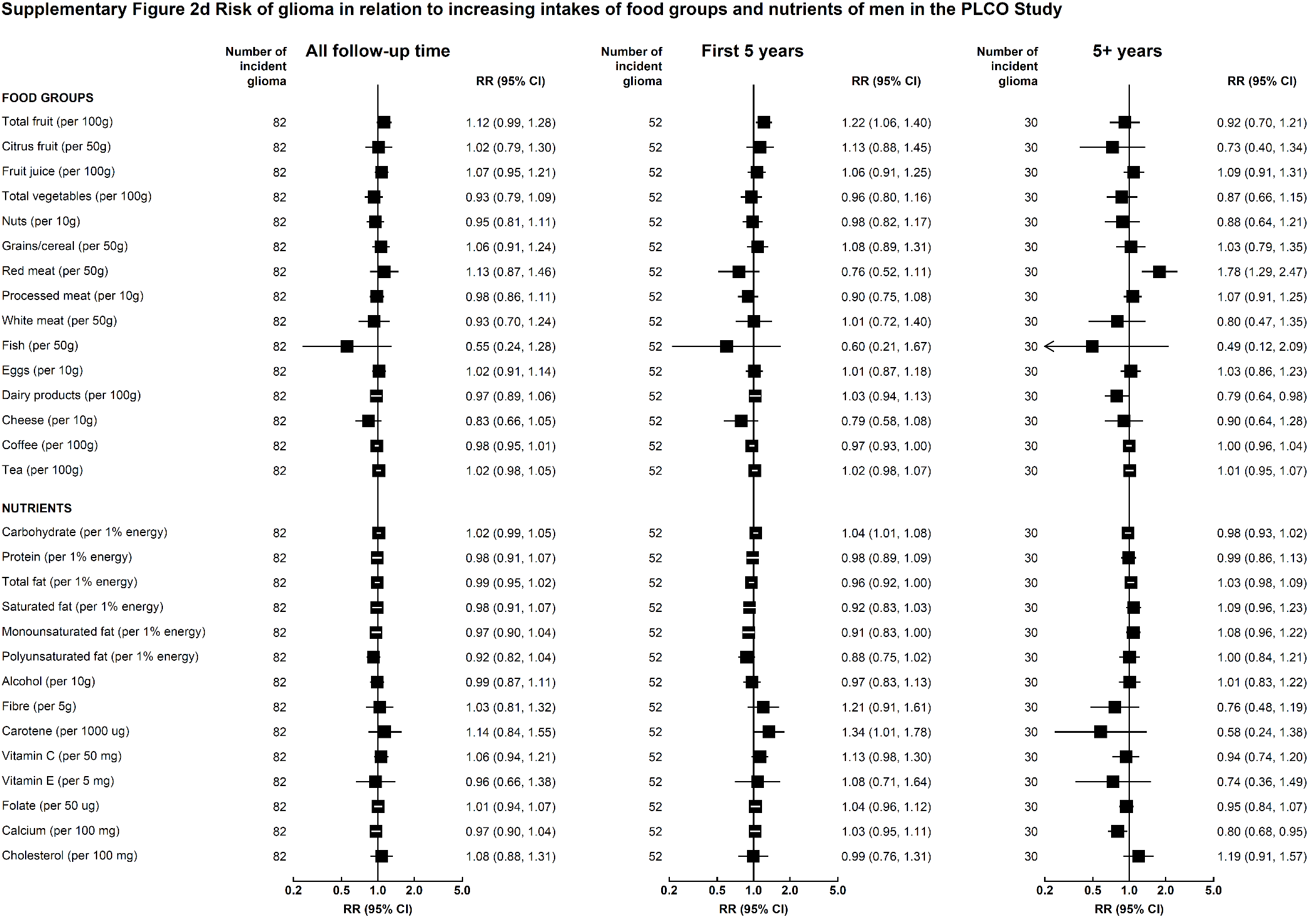


**Supplementary Figure 2e** Risk of glioma in relation to increasing intakes of food groups and nutrients of women in the PLCO Study


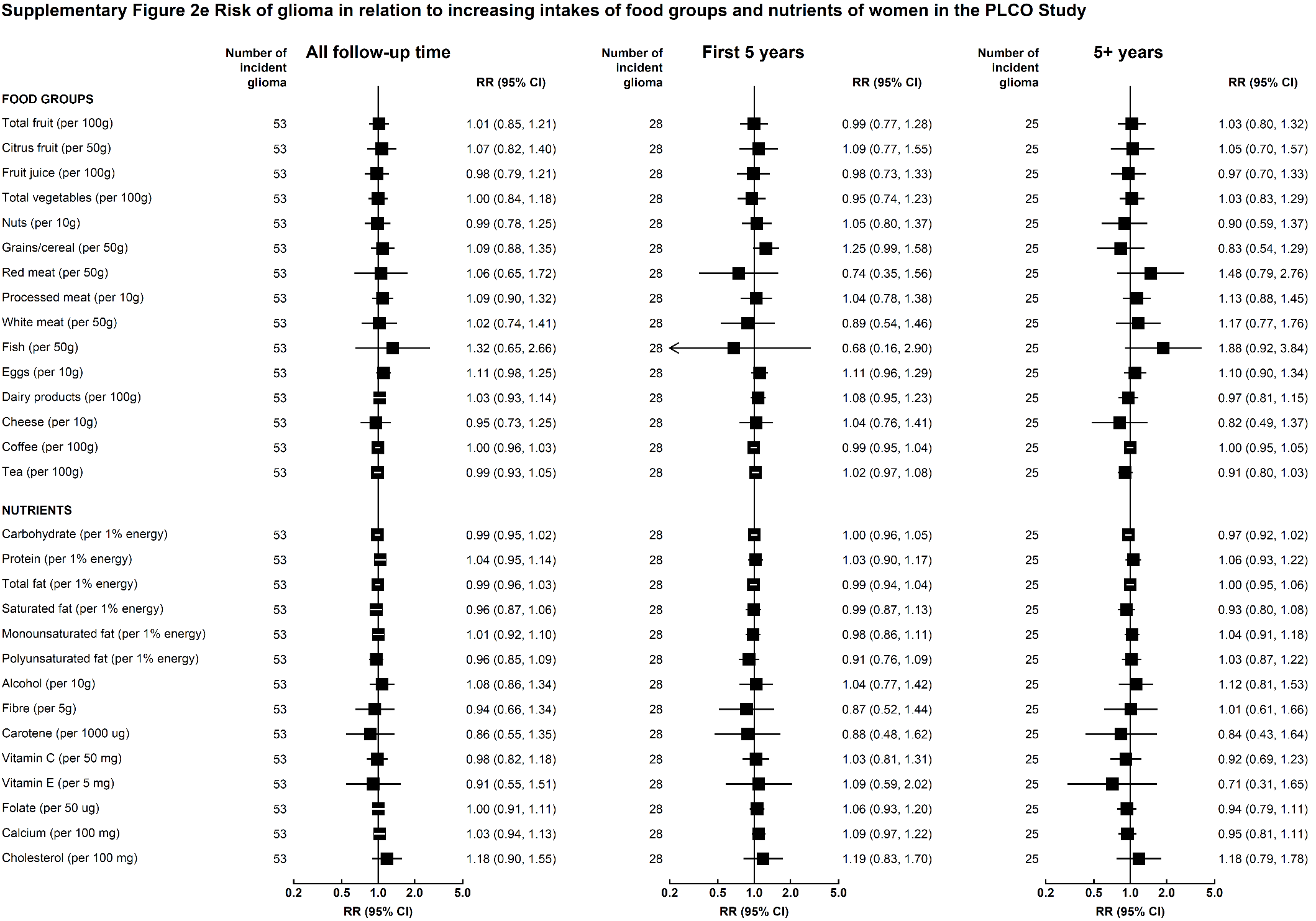


**REFERENCES**

**1.** Green J, Reeves GK, Floud S, et al. Cohort Profile: the Million Women Study. *Int J Epidemiol.* 2018.

**2.** Roddam AW, Spencer E, Banks E, et al. Reproducibility of a short semi-quantitative food group questionnaire and its performance in estimating nutrient intake compared with a 7-day diet diary in the Million Women Study. *Public Health Nutr.* 2005;8(2):201-213.

**3.** Liu B, Young H, Crowe FL, et al. Development and evaluation of the Oxford WebQ, a low-cost, web-based method for assessment of previous 24 h dietary intakes in large-scale prospective studies. *Public Health Nutr.* 2011;14(11):1998-2005.

**4.** ISD Scotland. Cancer Registry in Scotland. 2016 data completeness. http://www.isdscotland.org/Health-Topics/Cancer/Scottish-Cancer-Registry/. Accessed 5 June, 2018.

**5.** Office for National Statistics. Statistical bulletin: Cancer registration statistics, England: 2016. https://www.ons.gov.uk/peoplepopulationandcommunity/healthandsocialcare/conditionsanddiseases/bulletins/cancerregistrationstatisticsengland/final2016. Accessed 5 June, 2018.

**6.** Schatzkin A, Subar AF, Thompson FE, et al. Design and serendipity in establishing a large cohort with wide dietary intake distributions : the National Institutes of Health-American Association of Retired Persons Diet and Health Study. *Am J Epidemiol.* 2001;154(12):1119-1125.

**7.** Thompson FE, Kipnis V, Midthune D, et al. Performance of a food-frequency questionnaire in the US NIH-AARP (National Institutes of Health-American Association of Retired Persons) Diet and Health Study. *Public Health Nutr.* 2008;11(2):183-195.

**8.** United States Environmental Protection Agency. Analysis of Total Food Intake and Composition of Individual's Diet Based on the U.S. Department of Agriculture's 1994-96, 1998 Continuing Survey of Food Intakes by Individuals (CSFII) (2005, Final Report). Washington, DC, EPA/600/R-05/062F, United States Environmental Protection Agency, 2005.

**9.** Michaud D, Midthune D, Hermansen S, et al. Comparison of cancer registry case ascertainment with SEER estimates and self-reporting in a subset of the NIH-AARP Diet and Health Study. *J Registry Manag.* 2005;32:70–75.

**10.** Prorok PC, Andriole GL, Bresalier RS, et al. Design of the Prostate, Lung, Colorectal and Ovarian (PLCO) Cancer Screening Trial. *Control Clin Trials.* 2000;21(6 Suppl):273s-309s.

**11.** National Cancer Institute. The Prostate, Lung, Colorectal, and Ovarian Cancer Screening Trial. 2018; https://biometry.nci.nih.gov/cdas/plco/. Accessed 5 June, 2018.

**12.** Fung TT, Chiuve SE, McCullough ML, Rexrode KM, Logroscino G, Hu FB. Adherence to a DASH-style diet and risk of coronary heart disease and stroke in women. *Arch Intern Med.* 2008;168(7):713-720.

**13.** Trichopoulou A, Costacou T, Bamia C, Trichopoulos D. Adherence to a Mediterranean diet and survival in a Greek population. *N Engl J Med.* 2003;348(26):2599-2608.

**14.** Fung TT, Hu FB, Wu K, Chiuve SE, Fuchs CS, Giovannucci E. The Mediterranean and Dietary Approaches to Stop Hypertension (DASH) diets and colorectal cancer. *Am J Clin Nutr.* 2010;92(6):1429-1435.

**15.** Fung TT, McCullough ML, Newby PK, et al. Diet-quality scores and plasma concentrations of markers of inflammation and endothelial dysfunction. *Am J Clin Nutr.* 2005;82(1):163-173.

**16.** Guenther PM, Casavale KO, Reedy J, et al. Update of the Healthy Eating Index: HEI-2010. *J Acad Nutr Diet.* 2013;113(4):569-580.

**17.** Chiuve SE, Fung TT, Rimm EB, et al. Alternative dietary indices both strongly predict risk of chronic disease. *J Nutr.* 2012;142(6):1009-1018.
